# Diffusion of a disordered protein on its folded ligand

**DOI:** 10.1073/pnas.2106690118

**Published:** 2021-09-09

**Authors:** Felix Wiggers, Samuel Wohl, Artem Dubovetskyi, Gabriel Rosenblum, Wenwei Zheng, Hagen Hofmann

**Affiliations:** ^a^Department of Chemical and Structural Biology, Weizmann Institute of Science, 76100 Rehovot, Israel;; ^b^Department of Physics, Arizona State University, Tempe, AZ 85287;; ^c^College of Integrative Sciences and Arts, Arizona State University, Mesa, AZ 85212

**Keywords:** IDP, single-molecule FRET, protein dynamics, fuzzy complex, molecular simulation

## Abstract

Intrinsically disordered proteins often form dynamic complexes with their ligands. Yet, the speed and amplitude of these motions are hidden in classical binding kinetics. Here, we directly measure the dynamics in an exceptionally mobile, high-affinity complex. We show that the disordered tail of the cell adhesion protein E-cadherin dynamically samples a large surface area of the protooncogene β-catenin. Single-molecule experiments and molecular simulations resolve these motions with high resolution in space and time. Contacts break and form within hundreds of microseconds without a dissociation of the complex. The energy landscape of this complex is rugged with many small barriers (3 to 4 *k*_B_*T*) and reconciles specificity, high affinity, and extreme disorder. A few persistent contacts provide specificity, whereas unspecific interactions boost affinity.

## Significance

Flexibility in complexes between intrinsically disordered proteins and folded ligands is widespread in nature. However, timescales and spatial amplitudes of such dynamics remained unexplored for most systems. Our results show that the disordered cytoplasmic tail of the cell adhesion protein E-cadherin diffuses across the entire surface of its folded binding partner β-catenin at fast submillisecond timescales. The nanometer amplitude of these motions could allow kinases to access their recognition motifs without requiring a dissociation of the complex. We expect that the rugged energy landscape found in the E-cadherin/β-catenin complex is a defining feature of dynamic and partially disordered protein complexes.

Specific molecular interactions orchestrate a multitude of simultaneous cellular processes. The discovery of intrinsically disordered proteins (IDPs) (1, 2) has substantially aided our understanding of such interactions. More than two decades of research revealed a plethora of functions and mechanisms (2–6) that complemented the prevalent structure-based view on protein interactions. Even the idea that IDPs always ought to fold upon binding has largely been dismantled by recent discoveries of high-affine–disordered complexes (7, 8). Classical shape complementary is indeed superfluous in the complex between prothymosin-α and histone H1, in which charge complementary is the main driving force for binding (7). However, complexes between IDPs and folded proteins can also be highly dynamic [e.g., Sic1 and Cdc4 (9), the Na^+^/H^+^ exchanger tail and ERK2 (10), nucleoporin tails, and nuclear transport receptors (11)]. Yet timescales of motions and their spatial amplitudes are often elusive, such that it is unclear how precisely the surfaces of folded proteins alter the dynamics of bound IDPs. Answering this question is a key step in understanding how specificity, affinity, and flexibility can be simultaneously realized in such complexes.

**Fig. 1.**
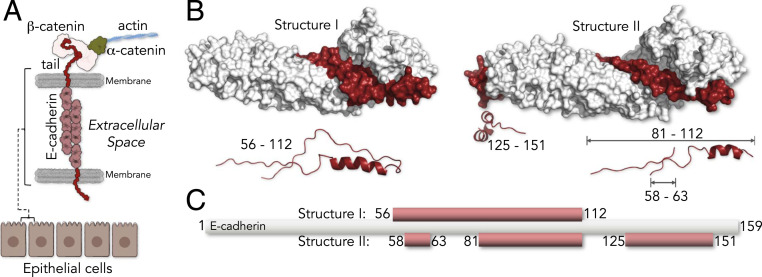
Complex between the cytoplasmic tail of E-cad and β-cat. (*A*) Schematics of cell–cell junctions mediated by E-cad and β-cat. (*B*) The two X-ray structures of the complex between the tail of E-cad (red) and the central repeat domain of β-cat (white) resolve different parts of E-cad (Protein Data Bank: 1i7x), indicating the flexibility of E-cad in the complex. (*Bottom*) Cartoon representation of the resolved E-cad parts. (*C*) Scheme showing the resolved parts of E-cad (red).

To address this question, we focused on the dynamics of the cell adhesion complex between E-cadherin (E-cad) and β-catenin (β-cat), which is involved in growth pathologies and cancer (12). E-cad is a transmembrane protein that mediates cell–cell adhesions by linking actin filaments of adjacent epithelial cells (Fig. 1*A*). Previous NMR results showed that the cytoplasmic tail of E-cad is intrinsically disordered (13). E-cad binds β-cat, which establishes a connection to the actin-associated protein α-catenin (14–16). β-cat, on the other hand, is a multifunctional repeat protein (17–20) that mediates cadherin-based cell adhesions (21) and governs cell fate decisions during embryogenesis (22). It contains three domains: an N-terminal domain (130 amino acids [aa]), a central repeat domain (550 aa), and a C-terminal domain (100 aa). Whereas the N- and C-terminal domains of β-cat are in large parts unstructured (17), with little effect on the affinity of the E-cad/β-cat complex (23), the 12 repeats of the central domain arrange in a superhelix (24). The X-ray structure showed that the E-cad wraps around this central domain of β-cat (24) (Fig. 1*B*). However, not only is half of the electron density of E-cad missing, the X-ray unit cell also comprises two structures with different resolved parts of E-cad (Fig. 1*B*). In fact, only 45% of all resolved E-cad residues are found in both structures (Fig. 1*C*). Although this ambiguity together with the large portion of missing residues (25) suggests that E-cad is highly dynamic in the complex with β-cat, the timescales and amplitudes of these dynamics are unknown.

Here, we integrated single-molecule Förster resonance energy transfer (smFRET) experiments with molecular simulations to directly measure the dynamics of E-cad on β-cat with high spatial and temporal resolution. In our bottom-up strategy, we first probed intramolecular interactions within E-cad using smFRET to parameterize a coarse-grained (CG) model. In a second step, we monitored E-cad on β-cat, integrated this information into the CG model, and obtained a dynamic picture of the complex. We found that all segments of E-cad diffuse on the surface of β-cat at submillisecond timescales and obtained a residue-resolved understanding of these motions: A small number of persistent interactions provide specificity, whereas many weak multivalent contacts boost affinity, which confirms the idea that regulatory enzymes access their recognition motifs on E-cad and β-cat without requiring the complex to dissociate (24).

## E-cad Is Expanded in Solution

**Fig. 2.**
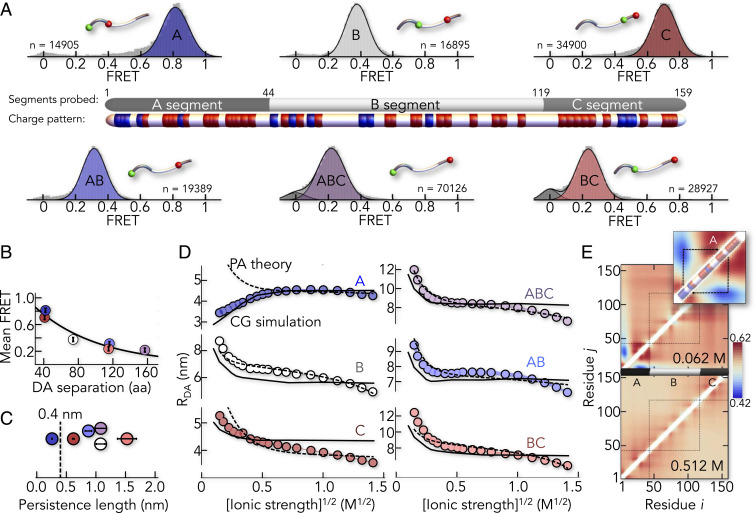
E-cad is expanded in solution. (*A*) SmFRET histograms of six segments of E-cad (A, B, C, AB, BC, and ABC) with *n* indicating the total number of molecules. A scheme shows the division of E-cad into the segments probed by FRET and the distribution of positively (blue) and negatively (red) charged residues. Histogram insets indicate the labeling sites as green and red spheres for donor and acceptor, respectively. (*B*) The mean FRET efficiencies scale with the sequence separation of the dyes. The color code is identical to *A*. (*C*) Persistence lengths of the individual segments. Error bars are the uncertainty introduced by the model of the distance distribution used (*SI Appendix*). (*D*) Salt-induced change in the DA distance, *R*_*DA*_, of the six E-cad segments (*SI Appendix*). A fit with the polyampholyte theory (30, 31) (dashed line) and the best fit of the CG model (solid line) are shown for comparison. (*E*) Length-scaling exponent map resulting from the best-fit CG model at low- (62 mM, *Upper*) and high- (512 mM, *Lower*) ionic strength. (*Inset*) A-segment.

To probe the conformation of E-cad, we labeled different regions with AlexaFluor 488 as donor and AlexaFluor 594 as acceptor. We created three constructs in which the FRET labels map the N-terminal (A), central (B), and C-terminal (C) segments of E-cad (Fig. 2*A* and *SI Appendix*, Table S1). In addition, we also checked the donor–acceptor (DA) distance of the longer segments AB, BC, and ABC to characterize the global behavior of E-cad. The labeled E-cad constructs were monitored while freely diffusing through the confocal volume of our microscope. For all six constructs, we obtained homogeneous FRET distributions (Fig. 2*A*) with mean positions that scaled with the sequence separation between the dyes (Fig. 2*B*), as expected for an IDP. The persistence lengths of all segments, except of the A-segment, significantly exceeded the value of 0.4 nm found for other disordered or unfolded proteins (26) (Fig. 2*C*). Hence, E-cad is expanded in solution, likely due to electrostatic repulsions given its high net charge (−22) (27–29). When we screened these repulsions with KCl, constructs ABC, AB, and BC collapsed substantially, a feature that is well described by mean-field polyampholyte theory (30) (Fig. 2*D*). Yet compaction was not uniform across the chain. Whereas the local segments B and C also compacted, the N-terminal A-segment expanded (Fig. 2*D*). Such chain expansion upon charge screening is known for polyampholytes with balanced numbers of positive and negative charges (29–33). However, the A-segment is not charge balanced. It even has a more negative net charge per residue (−0.186) than B (−0.093) and C (−0.166), thus excluding polyampholyte effects (30). Instead, the A-segment exhibits significant charge segregation: A positively charged N terminus (NT) is followed by a negative C-terminal part (Fig. 2*A*). Such charge patterning has previously been shown to compact disordered proteins (34), which we confirmed using a sequence charge decoration metric (SCD_lowsalt_) (35, 36) (*SI Appendix*, Fig. S1*A*). Mean-field polyampholyte theory is therefore inappropriate to describe E-cad, despite its success for other IDPs (29, 31) (Fig. 2*D*). To convert our smFRET experiments into structural ensembles, we modeled E-cad as a CG heteropolymer of beads. Each bead corresponded to an amino acid with charge (+1, 0, and −1) and hydrophobicity. The strength of hydrophobic interactions was adjustable via a global parameter ε (*SI Appendix*). Remarkably, a single value (ε = 0.16 kcal/mol) reproduced all experimental data, including those for the A-segment (Fig. 2*D*). A two-dimensional map of length scaling exponents (ν) for individual residue pairs (37) now clearly uncovers the effect of charge segregation in the A-segment (Fig. 2*E*). The scaling exponents for residue pairs from the positively charged NT and the negatively charged C terminus (CT) of the A-segment are small (ν < 0.5), which suggested strong attractions between the termini. Swapping a pair of charged residues between the termini would therefore revert the salt dependence of the A-segment (*SI Appendix*, Fig. S1*B*). Notably, high-salt concentrations (>500 mM) collapsed all segments (Fig. 2*D*), a salting-out effect (31) that is irrelevant at the physiological salt concentrations used here.

## E-cad Forms a Pliable Complex with β-cat

**Fig. 3.**
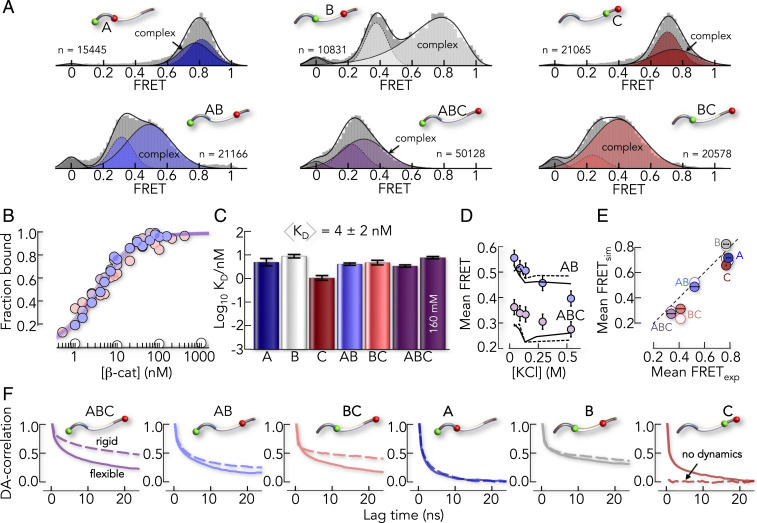
The conformation of E-cad in complex with β-cat is pliable. (*A*) smFRET histograms of the six E-cad constructs in the presence of β-cat (A: 4 nM, B: 85 nM, C: 15 nM, AB: 7 nM, BC: 8.5 nM, and ABC: 5 nM). Solid lines are fits of the histograms with a superposition of Gaussian and log-normal peaks and dashed lines represent free E-cad (*SI Appendix*). The number of molecules in each histogram is indicated by *n*. (*B*) Binding isotherms of selected E-cad segments (filled circles) and of the core-binding peptide (white circles). (*C*) *K*_*D*_ for the six constructs at 82 mM ionic strength and a comparison of the affinity of the ABC-segment at higher-ionic strength. (*D*) Change in FRET efficiency of the E-cad constructs ABC and AB in complex with β-cat, with increasing salt concentrations. Circles indicate the experimental data, and lines are the result from the rigid CG model when keeping the X-ray resolved E-cad parts static (dashed) and the best fit of the flexible CG model. (*E*) Comparison of the experimental FRET efficiencies with the rigid CG model (open circles) and the flexible CG model (filled circles). The dashed line is the identity line. (*F*) Normalized DA distance correlation functions from the rigid CG model (dashed lines) and the flexible CG model (solid lines).

To monitor E-cad in the complex, we added unlabeled β-cat, which gave rise to a second population of E-cad molecules with altered FRET efficiencies (Fig. 3*A*). In most segments, the FRET efficiency was higher than in free E-cad, indicating a compaction upon binding to β-cat. An exception was again the A-segment. The disruption of interactions between its oppositely charged termini slightly expanded this segment upon binding to β-cat. It has previously been shown that a correct charge patterning in the intrinsically disordered region of the Notch receptor has functional importance (38). The binding-induced breakage of charge contacts in the A-segment might have similar effects (e.g., exposing interaction sites for additional regulatory factors such as p120ctn) (39). We next performed smFRET experiments at different β-cat concentrations to determine the affinity of the complex (Fig. 3*B*). Depending on the segment, affinities range from 0.9 ± 0.1 nM (C-segment) to 7.6 ± 0.6 nM (B-segment) with an average of 4 ± 2 nM (Fig. 3*C*), which agrees with previous isothermal titration calorimetry results (23) (9.5 ± 3.2 nM at 23 °C; *SI Appendix*) and indicates that our FRET-dyes do not interfere with binding. Remarkably, whereas affinity was only moderately affected by salt (Fig. 3*C*), increasing KCl concentrations caused a significant decrease in the FRET efficiencies of E-cad, indicative of an expansion (Fig. 3*D*). This solvent sensitivity suggested that E-cad retains flexibility in complex with β-cat.

To understand this flexibility, we extended our CG model by intermolecular contacts between E-cad and the central armadillo repeat domain of β-cat (*SI Appendix*), neglecting the N- and C-terminal parts of β-cat for which structural information is lacking (17, 23, 24). We first tested a CG model, in which we rigidified the parts of E-cad that are resolved in the X-ray structures (24). Hence, structured parts of E-cad were essentially kept static while the unresolved parts were flexible but allowed to contact β-cat. Indeed, this “rigid” model described the observed FRET efficiencies within an error of ±0.07 FRET units (Fig. 3*E*). The model even partially captured the experimentally observed expansion of E-cad at higher-salt concentrations (Fig. 3*D*), suggesting that flexible parts are responsible for the effect. DA distance correlation functions, computed from microsecond simulations of our CG model, showed extensive decays in all segments except the C-segment, which interacted rigidly with β-cat (Fig. 3*F*). Unfortunately, CG models average over many degrees of freedom, such as side chain and solvent motions, and therefore only provide an estimate of the dynamics at a reduced timescale, typically two to three orders of magnitude faster compared to all-atom simulations. Yet the model is ideally suited to identify the presence or absence of dynamics. To check the predictions of the CG model, particularly the identified rigidity of the C-segment of E-cad in complex with β-cat, we directly measured the dynamics of E-cad in complex with β-cat experimentally.

## E-cad Is Highly Dynamic in Complex with β-cat

**Fig. 4.**
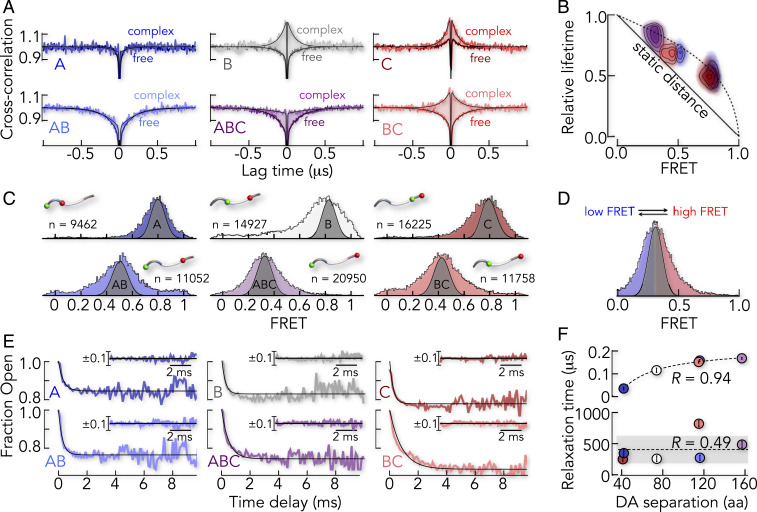
Timescales of motion of E-cad free and bound to β-cat. (*A*) DA cross-correlation functions of all six FRET constructs of E-cad in the absence (darker color) and presence (lighter color) of saturating amounts of β-cat (100 nM). Solid lines are fits to a product of exponential terms (*SI Appendix*). (*B*) Two-dimensional correlation map between the relative donor fluorescence lifetime ($\tau_{DA}/\tau_{D}$) and mean FRET efficiency of all six E-cad constructs in complex with β-cat. The solid line shows the expected dependence for a single DA distance. The dashed line indicates the dependence for a Gaussian chain distribution as an upper limit of distance heterogeneity. (*C*) FRET histograms of all E-cad segments in complex with β-cat, in comparison to the expected shot-noise limit obtained by recoloring (gray area). (*D*) FRET histogram of the E-cad ABC-segment, in which the low-FRET and high-FRET area is indicated in blue and red, respectively. RASP kinetics monitor the interconversion between the low-FRET molecules and high-FRET molecules. (*E*) RASP time decays of the fraction of molecules with FRET values lower than the mean value of the FRET histogram for all six E-cad constructs in complex with β-cat. Solid lines are exponential fits of the data. (*Inset*) Residuals of the exponential fit. (*F*) Comparison of the relaxation times of free E-cad (*Upper*) and β-cat–bound E-cad (*Lower*) as a function of the DA sequence separation. The Pearson correlation coefficient is indicated.

To probe the dynamics of the E-cad ensemble, we performed nanosecond fluorescence correlation spectroscopy (nsFCS) (40, 41) experiments of labeled E-cad in the absence and presence of β-cat. Disordered proteins such as E-cad exhibit large-scale distance fluctuations that cause anti-correlated DA intensity fluctuations (41). Indeed, the cross-correlation functions of free E-cad reveal anti-correlated decays in the 100 ns timescale for almost all segments (Fig. 4*A*). Only the C-segment lacks this signal, likely because of interdye contacts caused by a close proximity of the dyes in this short segment (*SI Appendix*, Fig. S2). Such dye–dye contacts quench both dyes simultaneously, thus causing positive amplitudes in the cross-correlation function.

When we formed the complex by adding β-cat, the correlation functions changed. Whereas signals in A and AB were nearly unaltered, the cross-correlation amplitude of the ABC segment decreased, indicating reduced fluctuations at the submicrosecond timescale (Fig. 4*A*). Segments B, C, and BC even showed positive cross-correlation amplitudes that cannot be explained by DA distance dynamics probed with FRET. A combination of increased static quenching due to dye contacts with aromatic residues of β-cat (42), but also restricted dye mobility (43), caused this behavior, as confirmed by control experiments using acceptor direct excitation (*SI Appendix*, Fig. S2) and fluorescence anisotropy measurements (*SI Appendix*, Fig. S3). To bypass these effects, we determined nanosecond donor fluorescence lifetimes for all segments, a quantity that is unaffected by static quenching, which occurs at hundreds of nanoseconds. If all members of an ensemble of E-cad/β-cat complexes obey the same DA distance, the donor fluorescence lifetime of this ensemble in the presence of acceptor ($\tau_{DA}$) is given by the measured FRET efficiency E via $\tau_{DA}=\tau_{D}\left(1-E\right)$ (41) (Fig. 4*B*). Here, $\tau_{D}$ is the donor fluorescence lifetime in the absence of the acceptor. This is not the case if the ensemble is heterogeneous (i.e., if DA distances differ among members of the ensemble) (*SI Appendix*). Indeed, we found substantial deviations from the linear scaling, suggesting an ensemble of structures. Importantly, we found this deviation also for the C-segment (Fig. 4*B*), which was not expected to be dynamic based on our CG model. In addition, we observed a substantial broadening of the FRET peaks of all segments in complex with β-cat, compared to the shot-noise–limited width obtained by recoloring (44) the data (Fig. 4*C*), which is symptomatic for dynamics in the millisecond regime. Several methods are available to identify dynamics at timescales between micro- and milliseconds for diffusing molecules, including burst variance analysis (45), probability distribution analysis (46, 47), Hidden Markov models (48), photon-by-photon likelihood approaches (49), and recurrence analysis of single particles (RASP) (50, 51). We chose RASP because of its potential to also quantify dynamics slower than the diffusion time of molecules through the confocal volume (∼1 ms) (50–52). The method exploits the fact that a diffusing molecule can enter and exit the confocal volume multiple times. Once a molecule leaves the observation volume, the chance of it returning within a short time interval is greater than the chance of detecting a new molecule (*SI Appendix*, Fig. S4). This effect allows us to obtain snapshots of the complex at timescales from 100 μs – 20 ms. By analyzing these events, we constructed the kinetics with which molecules switch from states with low FRET efficiency to those of high FRET efficiency and vice versa (Fig. 4*D* and *SI Appendix*). The resulting exchange kinetics indeed showed pronounced decays on timescales of micro- to millisecond for all six constructs (Fig. 4*E*), including the C-segment. The relaxation times obtained from exponential fits ranged from 250 ± 30 μs for the C-segment to 820 ± 50 μs for the BC-segment, i.e., three orders of magnitude slower than for free E-cad. Moreover, the relaxation times did not scale with the sequence separation of the dyes as expected for polymers (53–55) such as free E-cad (Fig. 4*F*). This showed that motions were determined by contacts between E-cad and β-cat rather than within E-cad. Most importantly, these experiments clearly showed that even the C-segment is highly dynamic. Yet, this finding does no necessarily disagree with the X-ray structure (24). In fact, the C-segment is only resolved in one of two structures in the unit cell (Fig. 1 *B* and *C*). Such ambiguity has also been observed in other complexes (25) and suggests that the C-segment can indeed dissociate from the β-cat surface, which explains the dynamics found experimentally. Hence, our results show that the complete cytoplasmic tail of E-cad samples the surface of β-cat at submillisecond to millisecond timescales. For comparison, the macroscopic dissociation rate of the complex is $k_{diss}=6.5\times 10^{-3}s^{-1}$ (23). Since the decay of macroscopic deviations from equilibrium for an ensemble is identical to the decay of the correlation of equilibrium fluctuations for a single molecule (56), the inverse macroscopic dissociation rate ($k_{diss}^{-1}=154\;s$) is identical to the average lifetime of an E-cad/β-cat complex. Given the timescale of equilibrium fluctuations of E-cad during this lifetime, we estimate that E-cad reconfigures more than 100,000 times before the complex dissociates.

## A Structural Model of E-cad in Complex with β-cat

**Fig. 5.**
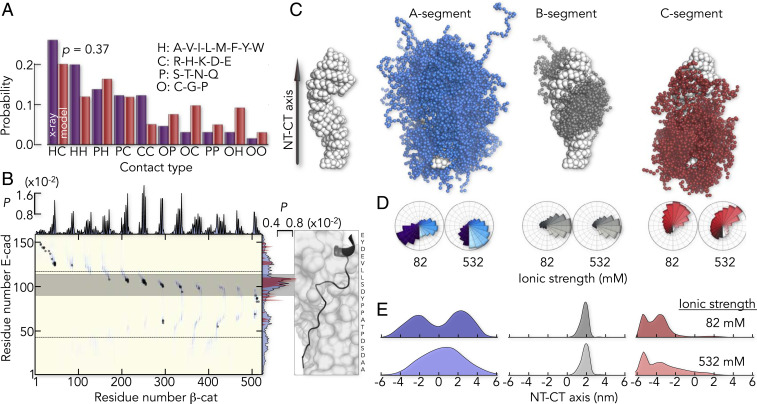
The flexible CG model predicts a heterogeneous, structural ensemble of E-cad in complex with β-cat. (*A*) Distribution of contact types in the complex. The classification of contacts is indicated. A Komolgorov–Smirnov test gives a probability of *P* = 0.37 that both distributions originate from the same sample. (*B*) Intermolecular contact probabilities (*P*) based on the flexible CG model (blue) overlaid with the contact map derived from the X-ray map (gray dots). The highest contact probabilities are found in a 20 amino acid long fragment in the B-segment (gray bar) that is well resolved in both X-ray structures (*Right*). The projected X-ray contact probabilities of E-cad, averaged over both X-ray structures and all β-cat residues (red area) are shown in comparison to the average contact probabilities of E-cad from the flexible CG model (blue area). (*C*) Structure of β-cat, indicating the directionality of the axis between (*Left*) and the ensemble of the center of mass positions (*Right*) of A- (blue), B- (gray), and C (red) -segment. (*D*) Center of mass angular distribution along the axis between the NT and CT of β-cat (schematically indicated) at low- (82 mM) and high- (532 mM) ionic strength. Color code is identical to *C*. (*E*) Center of mass positional distribution of E-cad projected onto the NT–CT axis of β-cat for the segments A, B, and C at two ionic strengths (indicated). The color code is identical to *D* and *C*.

To obtain a more realistic picture of the E-cad ensemble in complex with β-cat, we revised our CG-model. Instead of fully rigidifying the resolved parts of E-cad, we introduced a parameter (ζ) that tunes the strength of all intermolecular X-ray contacts in a global manner. We found that ζ = 0.6 kcal/mol described the experimentally determined FRET-values and the salt-sensitivity of the complex as good as the rigid model (Fig. 3 *D* and *E*). Most importantly, despite the fact that all X-ray contacts share the same interaction parameter ζ, the model indeed predicted a dynamic C-segment (Fig. 3*F*), which is in good accord with our experimental findings. To understand the deviations of this model from the known X-ray structure, we compared the distribution of contact types. To this end, we classified amino acids as polar (P), charged (C), hydrophobic (H), and other amino acids (O). Interestingly, the most abundant intermolecular contacts in the CG-model were of the uncommon H-C type instead of the commonly discussed C-C type (Fig. 5*A*), which explains the weak salt-dependence of the binding affinity (Fig. 3*C*). However, this distribution of contact types agrees well with the X-ray structure and an intermolecular contact map shows that interactions found in the X-ray structure were also preserved in the CG-model (Fig. 5*B*). Particularly, the residue-specific average contact probabilities of E-cad with β-cat in the CG-model, agree well with those computed from the two X-ray structures (Fig. 5*B*). Yet, the smFRET-based CG-model differed from the known structure in one important aspect. Contact probabilities (Fig. 5*B*) and lifetimes (*SI Appendix*, Fig. S5) were broadly distributed in the CG-model, reflecting many weak and unspecific interactions. The highest contact probabilities, i.e., the most persistent interactions, were found in a short 20 amino acid stretch of the B-segment (Fig. 5*B*), a region that is well resolved in both X-ray structures (Fig. 1 *B* and *C*). It has previously been identified as core-binding region of E-cad (57) and likely provides specificity. To disentangle contributions of specific and unspecific interactions to the overall stability of the E- cad/β-cat complex, we determined its affinity to β-cat (Fig. 3*B*). We found a low affinity of the core-binding region with an upper limit of disassociation constants (*K*_*D*_) > 1 μM compared to *K*_*D*_ ∼4 nM for full-length E-cad. Hence, flexible segments of E-cad boost affinity by more than 5.5 *k*_B_*T, *where *k*_B_ is the Boltzmann constant and *T* is the temperature. How are these segments distributed on the surface of β-cat?

The three-dimensional distribution of E-cad on β-cat differed strongly between segments (Fig. 5*C*). For instance, not only did the A-segment contact the NT and the CT of the central β-cat armadillo repeat domain, but also, the C-segment explored a large surface area of β-cat. The ensemble of the B-segment, on the other hand, was more confined, as expected from the higher-contact probabilities and the fact that 42% of it is resolved in both X-ray structures (Fig. 1*C*). We visualized the ensembles of the three segments by the distribution of their center of mass along two coordinates: an angular coordinate describing the distribution around the long axis of β-cat (Fig. 5*D*) and an axial coordinate for the distribution along β-cat (Fig. 5*E*). These distributions showed that the observed salt-induced changes of E-cad in complex with β-cat (Fig. 3*D*) were predominantly due to a screening of intra- and intermolecular charge contacts of the A-segment (Fig. 5 *D* and *E*), whereas changes in the B- and C-segment were much less pronounced.

In summary, the refined, flexible CG model indeed recapitulated the experimentally observed large-scale motions of E-cad on the β-cat surface. A total of 91% of the β-cat surface was explored by E-cad in a rather diffusive (i.e., continuous) manner, without encountering major barriers. However, given the simplification inherent to CG models, it remains to be determined whether a continuous diffusion of E-cad on the β-cat surface is realistic. An alternative model requires the concerted association and dissociation of E-cad segments, thus leading to dominant barriers that must be overcome to reconfigure on the β-cat surface. In fact, such a scenario has recently been suggested for the complex between β-cat and another ligand (TCF7L2) (58) and would explain why E-cad motions in the complex are orders of magnitude slower than those found for free E-cad (Fig. 4*F*). We therefore aimed at identifying the magnitude of these barriers.

## Intrachain Diffusion of E-cad on β-cat

**Fig. 6.**
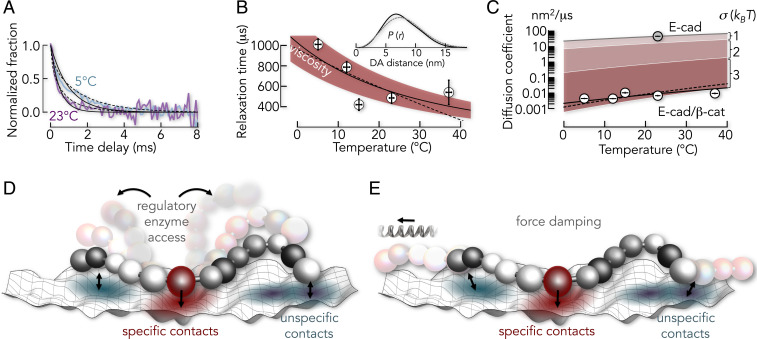
E-cad dynamics in complex with β-cat are diffusive. (*A*) Normalized time decays of the E-cad ABC construct at two temperatures (indicated). Solid and dashed lines are fits with an exponential function and with the Smoluchowski equation, respectively. (*B*) Relaxation times of the E-cad ABC construct as a function of temperature. The solid line is the dependence without activation energy (*SI Appendix*). The red area indicates the 90% confidence interval of this fit. The dashed line is an Arrhenius fit. (*Inset*) DA distance distributions for E-cad bound (black) and free (gray) from smFRET-based CG model. (*C*) Comparison of the diffusion coefficient of E-cad free (gray circle) and bound to β-cat (white circles). Shaded areas represent the effect of different roughness values (σ in *k*_B_*T*). Black solid and dashed lines are fits with the Zwanzig expressions (60) for periodic- (σ = 4.3 ± 0.1) and Gaussian- (σ = 2.9 ± 0.1) modulated roughness, respectively. (*D*) Scheme of E-cad on the surface of β-cat. Parts of E-cad can partially detach, thus giving access to regulatory enzymes. (*E*) Same as *D* but illustrating the spring-like properties of the E-cad/β-cat complex.

The experimental RASP kinetics of the six constructs agreed with exponential decays (Fig. 4*E*), a hallmark of two-state kinetics and indicative of a dominant barrier. We tested this hypothesis by monitoring the motions of E-cad ABC at different temperatures (*SI Appendix*, Fig. S6). A barrier should cause Arrhenius behavior (i.e., a significant slowdown of the dynamics with decreasing temperature). Yet we only found a twofold slowdown of the kinetics in the range from 23 to 5 °C (Fig. 6*A*). A fit with the Arrhenius equation $\tau =A\exp\left(\beta E_{a}\right)$ with $\beta^{-1}=k_{B}T$ yielded an activation energy of *E*_*a*_ = 28 ± 16 kJ/mol (Fig. 6*B*). However, this analysis neglects that the preexponential factor *A* depends on the viscosity of the medium (60), which itself scales with temperature. Accounting for this effect, we found that the slowdown was also reproduced without activation energy (Fig. 6*B*). We therefore checked whether the kinetics were indeed consistent with single-well (i.e., barrier-less diffusion), as suggested by our CG model. To this end, we used the DA distance distribution P(r) for the ABC construct from our flexible CG model (Fig. 6*B*, *Inset*) to fit the experimental RASP kinetics by solving the Smoluchowsky equation for diffusion in a single-well potential given by V(r)=−ln⁡P(r). Indeed, this barrier-less model also resulted in excellent fits. Intrachain diffusion coefficients ranged from (4.4 ± 0.1) × 10^−3^ nm^2^/μs to (9.5 ± 0.1) × 10^−3^ nm^2^/μs in the temperature range from 5 to 37 °C (Fig. 6*C* and *SI Appendix*). Notably, intrachain diffusion was four orders of magnitude slower than that of free E-cad ABC (42 ± 1 nm^2^/μs; *SI Appendix*), despite the absence of a dominant barrier (Fig. 6*C*).

Ruggedness in energy landscapes (i.e., a plethora of small barriers) is known to slow down motions as effectively as a dominant barrier (59, 61–63). For the case of one-dimensional diffusion in a potential of mean force, Zwanzig (59) derived expressions for the apparent diffusion coefficient (D) 1) for Gaussian-distributed potential wells with average depth σ, leading to super-Arrhenius behavior $D=D_{0}e^{-\beta^{2}\sigma^{2}}$ and 2) for a periodic modulation, leading to $D=D_{0}e^{-2\beta \sigma }$. Interestingly, the more general case of diffusion in multiple dimensions also leads to super-Arrhenius behavior (64). Here, $D_{0}$ and D are diffusion coefficients in the absence and presence of ruggedness, respectively. Experimentally, it has been challenging to obtain absolute values for σ, because $D_{0}$ and D are typically not simultaneously known. Our experiments allowed us to overcome this problem. Since the DA distance distribution of E-cad ABC changed only slightly upon binding to β-cat (Fig. 6 *B*, *Inset*), the free energy potentials obtained from the smFRET-based CG model were very similar between free and bound E-cad. Hence, $D_{0}$ and D were simply the diffusion coefficients of isolated E-cad and bound to β-cat, respectively. Using the above relationships, we found the amplitude to be 2.9 ± 0.1 and 4.3 ± 0.1 (in *k*_B_*T*) for randomly and periodically fluctuating ruggedness, respectively. These numbers suggest that the individual E-cad segments diffuse on a frustrated energy landscape with many local barriers of height 3 to 4 *k*_B_*T*.

## Discussion

A large number of disordered complexes, sometimes being referred to as “fuzzy complexes,” have been identified in the past (65, 66). The thermodynamic benefits of structural ambiguity, such as improved affinities due to higher-conformational entropy of the complex (67), increased specificity via interactions to very distant regions of a ligand (68) or facilitated binding via additional weak and transient interactions (4, 69), are also accompanied by functional benefits. For example, multisite phosphorylation gradually tunes the affinity of Sic1 for Cdc4 (9) and a similar mechanism had earlier also been suggested for E-cad and β-cat (24). Yet, compared to our understanding of the stability of disordered complexes, little is known about the speed at which disordered proteins reconfigure on the surface of their folded ligands. In fact, these dynamics are often only accessible in simulations (70). Our experiments showed that the reconfiguration of E-cad in complex with β-cat occurs at timescales from hundreds of microseconds to milliseconds (i.e., extremely fast compared to the timescales of association and dissociation but several orders of magnitude slower than the dynamics of unbound disordered E-cad). Clearly, the slower dynamics in the complex are caused by contacts with the protein surface. Yet these contacts do not result in a dominant free energy barrier that separates one conformation from the other. Instead, a large number of weak and transient contacts generate a rugged energy landscape of E-cad with many shallow minima. The minima depth (i.e., the average strength of these contacts) is in the order of a hydrogen bond (3 to 4 *k*_B_*T*) (71), which is insufficient to rigidify E-cad but sufficient to generate high affinity. Hence, similar to Sic1 and Cdc4, the flexible parts of E-cad serve an important function: they boost affinity. This is necessary because specific contacts of the core-binding region fail to generate sufficient binding strength, as indicated by the low affinity of the core-binding region of E-cad (Fig. 3*B*). However, the correct register of this element in the binding groove of β-cat provides specificity, which sets this complex apart from fully disordered complexes (7, 8).

Apart from allowing a gradual tuning of the E-cad/β-cat affinity via phosphorylation, the flexibility of E-cad in complex with β-cat might also allow kinases to access their recognition sites without dissociating the entire complex (72). Although the known association rates (3.5 × 10^5^ M^−1^ ⋅ s^−1^) (23) in combination with the in-cell concentration of β-cat (∼1 μM) (73) would suggest that rebinding of β-cat occurs within seconds after dissociation, the concentration of β-cat in the cytosol can be much lower. In fact, activation of Wnt-signaling is associated with an accumulation of β-cat in the nucleus, such that cytosolic concentrations can drop to 25 nM (73). In such cases, rebinding of β-cat already requires hundreds of seconds. The continuous attachment and detachment of local E-cad segments bypasses this problem by providing easy access for regulatory enzymes without dissociating and reassociating the complex (Fig. 6*D*). In addition, the complex can also serve mechanical functions. Although α-catenin is known to be the primary sensor for mechanotransduction in the cell adhesion complex (74), the flexibility of E-cad in complex with β-cat might serve as an additional entropic spring that dampens rupture forces between epithelial cells. Many weak contacts to the β-cat surface can increase the spring constant without causing a rigid complex (*SI Appendix*). The E-cad/β-cat complex has, therefore, the potential to act as a tunable damper for mechanical forces between cells. Yet whether the complex also shows catch–bond behavior [i.e., an increased number of intermolecular contacts in the presence of force (75, 76)] is currently unclear.

The Velcro-like design of many weak contacts on top of a few persistent interactions reconciles three seemingly contradictory factors: specificity, high affinity, and flexibility. Given the simplicity and advantages of this design, together with the fact that partially disordered complexes are highly abundant (65), the mode of interaction and the timescales of reconfiguration in the E-cad/β-cat complex may be common in biology.

## Supplementary Material

Supplementary File

Supplementary File

## Data Availability

Source data shown in the figures are provided with this paper (Dataset S1). A custom Wolfram symbolic transfer protocol add-on for Mathematica (Wolfram Research), used for the analysis of single-molecule fluorescence data, and implementation of the CG hydropathy scale (HPS) model in HOOMD-Blue have been deposited in Bitbucket (https://schuler.bioc.uzh.ch/programs/ and https://bitbucket.org/jeetain/hoomd_slab_builder). All other study data are included in the article and/or supporting information.

## References

[1] B. Schuler., Binding without folding—The biomolecular function of disordered polyelectrolyte complexes. Curr. Opin. Struct. Biol. 60, 66–76 (2020). 31874413 10.1016/j.sbi.2019.12.006

[2] R. van der Lee., Classification of intrinsically disordered regions and proteins. Chem. Rev. 114, 6589–6631 (2014). 24773235 10.1021/cr400525m PMC4095912

[3] V. Csizmok, A. V. Follis, R. W. Kriwacki, J. D. Forman-Kay, Dynamic protein interaction networks and new structural paradigms in signaling. Chem. Rev. 116, 6424–6462 (2016). 26922996 10.1021/acs.chemrev.5b00548 PMC5342629

[4] Y. Levy, J. N. Onuchic, P. G. Wolynes, Fly-casting in protein-DNA binding: Frustration between protein folding and electrostatics facilitates target recognition. J. Am. Chem. Soc. 129, 738–739 (2007). 17243791 10.1021/ja065531n

[5] J.-Y. Kim, F. Meng, J. Yoo, H. S. Chung, Diffusion-limited association of disordered protein by non-native electrostatic interactions. Nat. Commun. 9, 4707 (2018). 30413699 10.1038/s41467-018-06866-y PMC6226484

[6] J.-Y. Kim, H. S. Chung, Disordered proteins follow diverse transition paths as they fold and bind to a partner. Science 368, 1253–1257 (2020). 32527832 10.1126/science.aba3854 PMC8320588

[7] A. Borgia., Extreme disorder in an ultrahigh-affinity protein complex. Nature 555, 61–66 (2018). 29466338 10.1038/nature25762 PMC6264893

[8] E. D. Holmstrom, Z. Liu, D. Nettels, R. B. Best, B. Schuler, Disordered RNA chaperones can enhance nucleic acid folding via local charge screening. Nat. Commun. 10, 2453 (2019). 31165735 10.1038/s41467-019-10356-0 PMC6549165

[9] T. Mittag., Dynamic equilibrium engagement of a polyvalent ligand with a single-site receptor. Proc. Natl. Acad. Sci. U.S.A. 105, 17772–17777 (2008). 19008353 10.1073/pnas.0809222105 PMC2582940

[10] R. Hendus-Altenburger., The human Na(+)/H(+) exchanger 1 is a membrane scaffold protein for extracellular signal-regulated kinase 2. BMC Biol. 14, 31 (2016). 27083547 10.1186/s12915-016-0252-7 PMC4833948

[11] S. Milles., Plasticity of an ultrafast interaction between nucleoporins and nuclear transport receptors. Cell 163, 734–745 (2015). 26456112 10.1016/j.cell.2015.09.047 PMC4622936

[12] R. Nusse, H. Clevers, Wnt/β-catenin signaling, disease, and emerging therapeutic modalities. Cell 169, 985–999 (2017). 28575679 10.1016/j.cell.2017.05.016

[13] A. H. Huber, D. B. Stewart, D. V. Laurents, W. J. Nelson, W. I. Weis, The cadherin cytoplasmic domain is unstructured in the absence of beta-catenin. A possible mechanism for regulating cadherin turnover. J. Biol. Chem. 276, 12301–12309 (2001). 11121423 10.1074/jbc.M010377200

[14] H. Aberle., Assembly of the cadherin-catenin complex in vitro with recombinant proteins. J. Cell Sci. 107, 3655–3663 (1994). 7706414 10.1242/jcs.107.12.3655

[15] M. Ozawa, H. Baribault, R. Kemler, The cytoplasmic domain of the cell adhesion molecule uvomorulin associates with three independent proteins structurally related in different species. EMBO J. 8, 1711–1717 (1989). 2788574 10.1002/j.1460-2075.1989.tb03563.x PMC401013

[16] D. L. Rimm, E. R. Koslov, P. Kebriaei, C. D. Cianci, J. S. Morrow, Alpha 1(E)-catenin is an actin-binding and -bundling protein mediating the attachment of F-actin to the membrane adhesion complex. Proc. Natl. Acad. Sci. U.S.A. 92, 8813–8817 (1995). 7568023 10.1073/pnas.92.19.8813 PMC41057

[17] A. H. Huber, W. J. Nelson, W. I. Weis, Three-dimensional structure of the armadillo repeat region of beta-catenin. Cell 90, 871–882 (1997). 9298899 10.1016/s0092-8674(00)80352-9

[18] J. Behrens., Functional interaction of beta-catenin with the transcription factor LEF-1. Nature 382, 638–642 (1996). 8757136 10.1038/382638a0

[19] H. Hoschuetzky, H. Aberle, R. Kemler, Beta-catenin mediates the interaction of the cadherin-catenin complex with epidermal growth factor receptor. J. Cell Biol. 127, 1375–1380 (1994). 7962096 10.1083/jcb.127.5.1375 PMC2120252

[20] J. Hülsken, W. Birchmeier, J. Behrens, E-cadherin and APC compete for the interaction with beta-catenin and the cytoskeleton. J. Cell Biol. 127, 2061–2069 (1994). 7806582 10.1083/jcb.127.6.2061 PMC2120290

[21] H. Aberle, H. Schwartz, H. Hoschuetzky, R. Kemler, Single amino acid substitutions in proteins of the armadillo gene family abolish their binding to alpha-catenin. J. Biol. Chem. 271, 1520–1526 (1996). 8576147 10.1074/jbc.271.3.1520

[22] B. T. MacDonald, K. Tamai, X. He, Wnt/β-catenin signaling: Components, mechanisms, and diseases. Dev. Cell 17, 9–26 (2009). 19619488 10.1016/j.devcel.2009.06.016 PMC2861485

[23] H.-J. Choi, A. H. Huber, W. I. Weis, Thermodynamics of β-catenin-ligand interactions: The roles of the N- and C-terminal tails in modulating binding affinity. J. Biol. Chem. 281, 1027–1038 (2006). 16293619 10.1074/jbc.M511338200

[24] A. H. Huber, W. I. Weis, The structure of the beta-catenin/E-cadherin complex and the molecular basis of diverse ligand recognition by beta-catenin. Cell 105, 391–402 (2001). 11348595 10.1016/s0092-8674(01)00330-0

[25] S. DeForte, V. N. Uversky, Resolving the ambiguity: Making sense of intrinsic disorder when PDB structures disagree. Protein Sci. 25, 676–688 (2016). 26683124 10.1002/pro.2864 PMC4815412

[26] H. Hofmann., Polymer scaling laws of unfolded and intrinsically disordered proteins quantified with single-molecule spectroscopy. Proc. Natl. Acad. Sci. U.S.A. 109, 16155–16160 (2012). 22984159 10.1073/pnas.1207719109 PMC3479594

[27] B. Y. Ha, D. Thirumalai, Conformations of a polyelectrolyte chain. Phys. Rev. A 46, R3012–R3015 (1992). 9908546 10.1103/physreva.46.r3012

[28] H. Hofmann, R. P. Golbik, M. Ott, C. G. Hübner, R. Ulbrich-Hofmann, Coulomb forces control the density of the collapsed unfolded state of barstar. J. Mol. Biol. 376, 597–605 (2008). 18164723 10.1016/j.jmb.2007.11.083

[29] S. Müller-Späth., From the cover: Charge interactions can dominate the dimensions of intrinsically disordered proteins. Proc. Natl. Acad. Sci. U.S.A. 107, 14609–14614 (2010). Correction in: *Proc. Natl. Acad. Sci. U.S.A.* **110**, 16693 (2013). 20639465 10.1073/pnas.1001743107 PMC2930438

[30] P. G. Higgs, J.-F. Joanny, Theory of polyampholyte solutions. J. Chem. Phys. 94, 1543–1554 (1991).

[31] R. Vancraenenbroeck, Y. S. Harel, W. Zheng, H. Hofmann, Polymer effects modulate binding affinities in disordered proteins. Proc. Natl. Acad. Sci. U.S.A. 116, 19506–19512 (2019). 31488718 10.1073/pnas.1904997116 PMC6765308

[32] A. Bhattacharjee, P. Kundu, A. Dua, Self-consistent theory of structures and transitions in weak polyampholytes. Macromol. Theory Simul. 20, 75–84 (2011).

[33] H. S. Samanta, D. Chakraborty, D. Thirumalai, Charge fluctuation effects on the shape of flexible polyampholytes with applications to intrinsically disordered proteins. J. Chem. Phys. 149, 163323 (2018). 30384718 10.1063/1.5035428

[34] R. K. Das, R. V. Pappu, Conformations of intrinsically disordered proteins are influenced by linear sequence distributions of oppositely charged residues. Proc. Natl. Acad. Sci. U.S.A. 110, 13392–13397 (2013). 23901099 10.1073/pnas.1304749110 PMC3746876

[35] L. Sawle, K. Ghosh, A theoretical method to compute sequence dependent configurational properties in charged polymers and proteins. J. Chem. Phys. 143, 085101 (2015). 26328871 10.1063/1.4929391

[36] J. Huihui, T. Firman, K. Ghosh, Modulating charge patterning and ionic strength as a strategy to induce conformational changes in intrinsically disordered proteins. J. Chem. Phys. 149, 085101 (2018). 30193467 10.1063/1.5037727

[37] G.-N. W. Gomes., Conformational ensembles of an intrinsically disordered protein consistent with NMR, SAXS, and single-molecule FRET. J. Am. Chem. Soc. 142, 15697–15710 (2020). 32840111 10.1021/jacs.0c02088 PMC9987321

[38] K. P. Sherry, R. K. Das, R. V. Pappu, D. Barrick, Control of transcriptional activity by design of charge patterning in the intrinsically disordered RAM region of the Notch receptor. Proc. Natl. Acad. Sci. U.S.A. 114, E9243–E9252 (2017). 29078291 10.1073/pnas.1706083114 PMC5676888

[39] T. Pieters, F. van Roy, J. van Hengel, Functions of p120ctn isoforms in cell-cell adhesion and intracellular signaling. Front. Biosci. (Landmark Ed) 17, 1669–1694 (2012). 22201829 10.2741/4012

[40] D. Nettels, I. V. Gopich, A. Hoffmann, B. Schuler, Ultrafast dynamics of protein collapse from single-molecule photon statistics. Proc. Natl. Acad. Sci. U.S.A. 104, 2655–2660 (2007). 17301233 10.1073/pnas.0611093104 PMC1815237

[41] B. Schuler, A. Soranno, H. Hofmann, D. Nettels, Single-molecule FRET spectroscopy and the polymer physics of unfolded and intrinsically disordered proteins. Annu. Rev. Biophys. 45, 207–231 (2016). 27145874 10.1146/annurev-biophys-062215-010915

[42] D. Haenni, F. Zosel, L. Reymond, D. Nettels, B. Schuler, Intramolecular distances and dynamics from the combined photon statistics of single-molecule FRET and photoinduced electron transfer. J. Phys. Chem. B 117, 13015–13028 (2013). 23718771 10.1021/jp402352s

[43] F. Hillger., Probing protein-chaperone interactions with single-molecule fluorescence spectroscopy. Angew. Chem. Int. Ed. Engl. 47, 6184–6188 (2008). 18618555 10.1002/anie.200800298

[44] I. V. Gopich, A. Szabo, Decoding the pattern of photon colors in single-molecule FRET. J. Phys. Chem. B 113, 10965–10973 (2009). 19588948 10.1021/jp903671p PMC2802060

[45] J. P. Torella, S. J. Holden, Y. Santoso, J. Hohlbein, A. N. Kapanidis, Identifying molecular dynamics in single-molecule FRET experiments with burst variance analysis. Biophys. J. 100, 1568–1577 (2011). 21402040 10.1016/j.bpj.2011.01.066 PMC3059737

[46] S. Kalinin, A. Valeri, M. Antonik, S. Felekyan, C. A. M. Seidel, Detection of structural dynamics by FRET: A photon distribution and fluorescence lifetime analysis of systems with multiple states. J. Phys. Chem. B 114, 7983–7995 (2010). 20486698 10.1021/jp102156t

[47] S. Kalinin, E. Sisamakis, S. W. Magennis, S. Felekyan, C. A. M. Seidel, On the origin of broadening of single-molecule FRET efficiency distributions beyond shot noise limits. J. Phys. Chem. B 114, 6197–6206 (2010). 20397670 10.1021/jp100025v

[48] M. Pirchi., Photon-by-photon hidden Markov model analysis for microsecond single-molecule FRET kinetics. J. Phys. Chem. B 120, 13065–13075 (2016). 27977207 10.1021/acs.jpcb.6b10726

[49] I. V. Gopich, A. Szabo, Theory of the energy transfer efficiency and fluorescence lifetime distribution in single-molecule FRET. Proc. Natl. Acad. Sci. U.S.A. 109, 7747–7752 (2012). 22550169 10.1073/pnas.1205120109 PMC3356627

[50] A. Hoffmann., Quantifying heterogeneity and conformational dynamics from single molecule FRET of diffusing molecules: Recurrence analysis of single particles (RASP). Phys. Chem. Chem. Phys. 13, 1857–1871 (2011). 21218223 10.1039/c0cp01911a PMC3378030

[51] I. Grossman-Haham, G. Rosenblum, T. Namani, H. Hofmann, Slow domain reconfiguration causes power-law kinetics in a two-state enzyme. Proc. Natl. Acad. Sci. U.S.A. 115, 513–518 (2018). 29298911 10.1073/pnas.1714401115 PMC5776979

[52] T. Lasitza-Male., Membrane chemistry tunes the structure of a peptide transporter. Angew. Chem. Int. Ed. Engl. 59, 19121–19128 (2020). 32744783 10.1002/anie.202008226 PMC7590137

[53] A. Soranno., Quantifying internal friction in unfolded and intrinsically disordered proteins with single-molecule spectroscopy. Proc. Natl. Acad. Sci. U.S.A. 109, 17800–17806 (2012). 22492978 10.1073/pnas.1117368109 PMC3497802

[54] A. Soranno, F. Zosel, H. Hofmann, Internal friction in an intrinsically disordered protein-comparing Rouse-like models with experiments. J. Chem. Phys. 148, 123326 (2018). 29604877 10.1063/1.5009286

[55] P. E. Rouse Jr, A theory of the linear viscoelastic properties of dilute solutions of coiling polymers. J. Chem. Phys. 21, 1272–1280 (1953).

[56] L. Onsager, Reciprocal relations in irreversible processes. I. Phys. Rev. 37, 405–426 (1931).

[57] J. Stappert, R. Kemler, A short core region of E-cadherin is essential for catenin binding and is highly phosphorylated. Cell Adhes. Commun. 2, 319–327 (1994). 7820535 10.3109/15419069409014207

[58] B. M. Smith, P. J. E. Rowling, C. M. Dobson, L. S. Itzhaki, Parallel and sequential pathways of molecular recognition of a tandem-repeat protein and its intrinsically disordered binding partner. Biomolecules 11, 827 (2021). 34206070 10.3390/biom11060827 PMC8228192

[59] R. Zwanzig, Diffusion in a rough potential. Proc. Natl. Acad. Sci. U.S.A. 85, 2029–2030 (1988). 3353365 10.1073/pnas.85.7.2029 PMC279921

[60] H. Kramers, Brownian motion in a field of force and the diffusion model of chemical reactions. Physica 7, 284–304 (1940).

[61] H. Frauenfelder, S. G. Sligar, P. G. Wolynes, The energy landscapes and motions of proteins. Science 254, 1598–1603 (1991). 1749933 10.1126/science.1749933

[62] A. Ansari., Protein states and proteinquakes. Proc. Natl. Acad. Sci. U.S.A. 82, 5000–5004 (1985). 3860839 10.1073/pnas.82.15.5000 PMC390486

[63] B. G. Wensley., Experimental evidence for a frustrated energy landscape in a three-helix-bundle protein family. Nature 463, 685–688 (2010). 20130652 10.1038/nature08743 PMC2851140

[64] J. D. Bryngelson, P. G. Wolynes, Intermediates and barrier crossing in a random energy model (with applications to protein folding). J. Phys. Chem. 93, 6902–6915 (1989).

[65] M. Fuxreiter, P. Tompa, Fuzzy complexes: A more stochastic view of protein function Adv. Exp. Med. Biol. 725, 1–14 (2012). 22399315 10.1007/978-1-4614-0659-4_1

[66] A. N. Amin, Y.-H. Lin, S. Das, H. S. Chan, Analytical theory for sequence-specific binary fuzzy complexes of charged intrinsically disordered proteins. J. Phys. Chem. B 124, 6709–6720 (2020). 32639157 10.1021/acs.jpcb.0c04575

[67] T. A. Graham, D. M. Ferkey, F. Mao, D. Kimelman, W. Xu, Tcf4 can specifically recognize beta-catenin using alternative conformations. Nat. Struct. Biol. 8, 1048–1052 (2001). 11713475 10.1038/nsb718

[68] C. Smet-Nocca, J.-M. Wieruszeski, V. Chaar, A. Leroy, A. Benecke, The thymine-DNA glycosylase regulatory domain: Residual structure and DNA binding. Biochemistry 47, 6519–6530 (2008). 18512959 10.1021/bi7022283

[69] A. Tóth-Petróczy, I. Simon, M. Fuxreiter, Y. Levy, Disordered tails of homeodomains facilitate DNA recognition by providing a trade-off between folding and specific binding. J. Am. Chem. Soc. 131, 15084–15085 (2009). 19919153 10.1021/ja9052784

[70] L. S. Bigman, Y. Levy, Tubulin tails and their modifications regulate protein diffusion on microtubules. Proc. Natl. Acad. Sci. U.S.A. 117, 8876–8883 (2020). 32245812 10.1073/pnas.1914772117 PMC7183165

[71] S.-Y. Sheu, D.-Y. Yang, H. L. Selzle, E. W. Schlag, Energetics of hydrogen bonds in peptides. Proc. Natl. Acad. Sci. U.S.A. 100, 12683–12687 (2003). 14559970 10.1073/pnas.2133366100 PMC240678

[72] J. Henriques, K. Lindorff-Larsen, Protein dynamics enables phosphorylation of buried residues in Cdk2/Cyclin-A-bound p27. Biophys. J. 119, 2010–2018 (2020). 33147476 10.1016/j.bpj.2020.06.040 PMC7732742

[73] C. W. Tan., Wnt signalling pathway parameters for mammalian cells. PLoS One 7, e31882 (2012). 22363759 10.1371/journal.pone.0031882 PMC3283727

[74] A. K. Barry., α-catenin cytomechanics–role in cadherin-dependent adhesion and mechanotransduction. J. Cell Sci. 127, 1779–1791 (2014). 24522187 10.1242/jcs.139014 PMC3986676

[75] S. Chakrabarti, M. Hinczewski, D. Thirumalai, Plasticity of hydrogen bond networks regulates mechanochemistry of cell adhesion complexes. Proc. Natl. Acad. Sci. U.S.A. 111, 9048–9053 (2014). 24927549 10.1073/pnas.1405384111 PMC4078813

[76] M. Dembo, D. Torney, K. Saxman, D. Hammer, The reaction-limited kinetics of membrane-to-surface adhesion and detachment. Proc. R Soc. Lond. Biol. Sci. B 234, 55–83 (1988). 10.1098/rspb.1988.0038 2901109

