# Supplementary material for: Diffusion of a disordered protein on its folded ligand
Source: Proc Natl Acad Sci U S A. 2021 Sep 9;118(37):e2106690118. doi: 10.1073/pnas.2106690118 (PMC8449409; doi:10.1073/pnas.2106690118)
Supplement: Supplementary File [file pnas.2106690118.sapp.pdf]

SI Appendix

Methods.

Constructs. The plasmid containing the cytoplasmic domain of mouse E-cad (residues 734-884, UniProt ID P09803) was a kind gift of William I. Weis from Stanford University School of Medicine. E-cad was transferred from the pGEX vector to a K151 vector system using transfer-PCR. The procedure was carried out as described previously (1). Additionally, cysteine linkers were incorporated in the E-cad-K151 vector using the restriction free cloning approach (2) at different positions to generate the six labeling variants (SI Appendix Table S1). Full-length murine-beta-catenin (UniProt ID Q02284, purchased from GenScript) was transferred to a K151 vector system using transfer-PCR. Both proteins start with an N-terminal His_{12}-tag followed by the Sumo protein.

Protein expression. Proteins were expressed in *E. coli* BL21(DE3) cells. A 50 ml overnight culture grown at 37 °C (100 µg ml^{-1} Kanamycin) was used to inoculate 4 l of super broth (100 µg ml^{-1} Kanamycin). The cultures were grown at 37 °C to an optical density of 0.6 - 0.8 cm^{-1} (at 600 nm). Then 0.5 mM isopropyl-β-D-thiogalactoside was added to induce protein expression for 3 h. After induction, the cultures were cooled to 4 °C and centrifuged at 3000 g for 30 min at 4 °C. The cell pellet was washed with 80 ml of ice-cold buffer (10 mM Tris-HCl pH 8.0, 300 mM NaCl) and centrifuged at 3000 g for 30 min at 4 °C. The supernatants were discarded and the pellets were stored at -80 °C.

Protein purification. The cell pellet from 2 l culture was re-suspended in 50 mM Tris-HCl pH 8.0, 100 mM NaCl, 10 % glycerol, 1 mM DTT, 0.5 mM phenyl-methanesulfonyl fluoride (PMSF), 5 µM leupeptin, 2.5 µg ml-1 pepstatin (25 ml per 5 g wet weight) followed by sonication on ice using an ultrasonicator (Vibra-Cell, Sonics) at 70 % amplitude for 5 times 30 s with a 2 s on / 8 s off pulse. The soluble fraction was collected by centrifugation at 40,000 g for 30 min at 4 °C. Following sonication, DNA impurities were digested (DNase: Fisher Optizyme, 2 U/ml) and the supernatant was filtered (0.45 µm, Sartorius) and loaded at 4 °C with a flow rate of 0.5 ml min^{-1} onto a 5 ml HisTrap HP column (GE Healthcare) equilibrated with 50 mM Tris-HCl pH 8.0, 100 mM NaCl, 10 % glycerol. The column was subsequently washed with 20 ml of the equilibration buffer followed by 20 ml of 50 mM Tris-HCl pH 8.0, 500 mM NaCl and with additional 20 ml of 50 mM Tris-HCl pH 8.0, 470 mM NaCl, 75 mM imidazole at a flow rate of 1 ml min^{-1}. The protein was eluted with 20 ml of 50 mM Tris-HCl pH 8.0, 300 mM NaCl, 500 mM imidazole at a flow rate of 1 ml min^{-1}. Protein fractions were pooled.

Removal of the His$_6$-Sumo tag. The affinity tags of both proteins were removed using Sumo protease, bdSENP1 (3). The plasmid was a kind gift of Professor Dirk Görlich, Max-Planck-Institute for Biophysical Chemistry (Göttingen, Germany). A solution containing 300 nM Sumo protease was dialyzed twice against 50 mM Tris-HCl pH 8.0, 500 mM NaCl for 2h at 4°C. Afterwards, the sample was loaded at 2 ml min^{-1} onto a 5 ml HisTrap HP column (GE Healthcare) equilibrated with 50 mM Tris-HCl pH 8.0, 470 mM NaCl, 75 mM imidazole at 4°C. The column was washed with 20 ml of the same buffer. The flow-through containing the cleaved protein was collected. The protease and non-cleaved proteins were eluted with 20 ml of buffer B at a flow rate of 2 ml min^{-1}. Protein fractions were pooled and submitted to size exclusion chromatography (SEC) using a Superdex 200 pg HiLoad 26/600 column (GE Healthcare Life Sciences) equilibrated with 20 mM Tris/HCl, 300 mM NaCl, 5 mM DTT, pH 8.0. Protein fractions were collected and pooled. Final refinement of the β-cat was conducted with a buffer exchange using a 5 ml GE, HiTrap desalting column into 25 mM Tris/HCl, 50 mM KCl, 200 mM L-Arg, pH 8, followed by concentrating the sample (VivaSpin 6 MWCO 3 kDa cut-off, GE Healthcare) to 8 µM. The E-cad constructs were concentrated to ~ 14 µM. Protein concentrations were determined based on the absorbance at 280 nm using the extinction coefficient at 280 nm of 18910 M^{-1}cm^{-1} for the E-cad variants and 63830 M^{-1}cm^{-1} for β-cat. All E-cad constructs were additionally purified using reverse-phase HPLC purification with the ZORBAX Eclipse Plus C18 (3.5 µm) column (Agilent) (see following section). The correct protein mass was confirmed via ESI mass spectrometry analysis. The proteins were drop-frozen in liquid nitrogen and stored at -80 °C.

Fluorophore labeling with AlexaFluor 488 (donor) and AlexaFluor 594 (acceptor). The six E-cad variants were reduced before labeling by the addition of 100 mM DTT. After 15 min at room temperature, the proteins were purified via reversed phase HPLC using a ZORBAX Eclipse Plus C18 (3.5 µm) column (Agilent) equilibrated with 0.1% trifluoroacetic acid (TFA). The E-cad constructs were eluted with gradients from 15% - 60% acetonitrile (ACN) in 13 ml. Purified proteins were lyophilized and stored at -80 °C. The lyophilized proteins were re-solubilized in 50 mM sodium phosphate pH 7.3, 6 M GdmCl at a protein concentration of 150 µM. The precise concentrations were determined using the absorbance at 280 nm. For labeling, the proteins were incubated with 0.6 equivalents of AlexaFluor 488 C5 maleimide (Invitrogen) for 60 min (25 °C, 300 rpm). Afterwards, a tenfold excess of β-mercaptoethanol (compared to the dye) was added to quench unreacted dye. Reversed-phase HPLC was then used to remove unreacted dye, unlabeled and double-donor labeled protein. To this end, a ZORBAX Eclipse Plus C18 (3.5 µm) column (Agilent) equilibrated with water containing 0.1 % TFA was used. The gradient was 38% - 46% ACN in 15 ml. The pooled fractions were lyophilized and stored at -80 °C. The donor-labeled protein was re-solubilized in the labeling buffer to obtain a protein

concentration of approximately 20 μM and the correct protein concentration was determined using the absorbance at 493 nm and the extinction coefficient of the Alexa 488 dye (72000 $M^{-1}cm^{-1}$). A 5-fold excess of acceptor dye Alexa 594 C5 maleimide (Invitrogen) was added and samples were incubated for 180 min at (25°C, 300 rpm). Afterwards, the reaction was quenched using β-mercaptoethanol. Again, reversed-phase HPLC was used to remove free label using the same column as described above. The doubly labeled proteins were eluted using with a 25% - 40% ACN gradient. The pooled fractions were lyophilized and stored at -80 °C. The correct labeling of all protein variants was confirmed using ESI mass spectrometry.

Single-molecule fluorescence spectroscopy. All single-molecule fluorescence experiments were performed with a MicroTime 200 confocal microscope (PicoQuant) equipped with an Olympus IX73 inverted microscope. We either used linearly polarized light from a 485 nm diode laser (LDH-D-C-485, PicoQuant) adjusted to 100 μW (measured at the back aperture of the objective) to excite the donor fluorophore with a repetition rate of 40 MHz or two pulsed lasers controlled by a PDL 828-L "Sepia II" (PicoQuant, Germany) for Pulsed Interleaved Excitation (PIE) experiments (4, 5). In PIE experiments, the interleaved excitation of the acceptor dye allows us to identify molecules carrying both donor and acceptor dyes. To this end, two light sources, a 485 nm diode laser (LDH-D-C-485, PicoQuant) and a white-light laser (Solea, PicoQuant) set to an excitation wavelength of 595 nm, were used to excite the donor and the acceptor dyes alternatingly at a total repetition rate per period of 20 MHz. The laser intensities were adjusted to 100 μW at 485 nm and 20 μW at 595 nm (Pm100D, Thor Labs). The excitation beam was guided through a major dichroic mirror (ZT 470-491/594 rpc, Chroma) to a 60x, 1.2NA water objective (Olympus) that focuses the beam into the sample. The sample was placed in a homemade cuvette (quartz 25 mm diameter round cover slips, Esco Optics), borosilicate glass 6 mm diameter cloning cylinder (Hilgenberg)) with a volume of 50 μl. Photons emitted from the sample were collected by the same objective and after passing the major dichroic mirror (ZT 470-491/594 rpc, Chroma), the residual excitation light was filtered by a long-pass filter (BLP01-488R, Semrock) and sent through a 100 μm pinhole to remove out-of-focus light. The sample fluorescence was detected with either two channels or four channels for fluorescence anisotropy measurements. Donor and acceptor fluorescence was separated via a dichroic mirror (T585 LPXR, Chroma) and each color was focused onto a single-photon avalanche diode (SPAD) (Excelitas) with additional bandpass filters: FF03-525/50, (Semrock) for the donor SPAD and FF02-650/100 (Semrock) for the acceptor SPAD. For fluorescence anisotropy measurements, the emission light was first separated into its parallel and perpendicular components with respect to the linearly polarized excitation light via a polarizing beam splitter and each component was separated by two dichroic mirrors into donor and acceptor photons resulting. The arrival time of every detected photon was recorded with a HydraHarp

400 time-correlated single photon counting (TCSPC) module (PicoQuant) and stored with a resolution of 8 ps (16 ps for PIE). The labeled proteins were diluted to a concentration of approximately 50 pM in 20 mM Tris-HCl pH 8.0 (12 mM ionic strength) at the appropriate concentrations of KCl in the absence or presence of unlabeled β-cat. To prevent surface adhesion of the proteins and to maximize photon emission, 0.001% Tween 20 (Pierce) and 20 mM DTT were included in the buffer. Measurements in the presence of β-cat contained in addition 20 mM L-Arg (for a total of 32 mM ionic strength together with the Tris-HCl buffer) to prevent aggregation. All measurements were performed at 23°C.

Single-molecule identification and FRET corrections. Instrumental imperfections and differences in the brightness of donor and acceptor require a correction of the detected raw photon counts (6, 7). Although many of the experiments were performed with four detection channels, two donor channels and two acceptor channels with different polarizations, we describe the molecule identification and corrections for a two-channel (channel 1 and 2) setup. However, the extension to four channels is straightforward. These corrections are defined by five parameters: γ_1 and γ_2 that account for the different detection probabilities of photons from the two dyes, β_{21} and β_{12}, the leakage of donor photons into the acceptor channel 1 and the leakage of acceptor photons into the donor channel 2, respectively, and α, the probability to directly excite the acceptor dye at the wavelength specific for the donor. If n_1 and n_2 are the detected photons in the acceptor and donor channels, respectively, and b_1 and b_2 are the background rates in both channels, the corrected photon counts for acceptor and donor (n_A' and n_D') are given by

$$\begin{pmatrix} n_A \\ n'_D \end{pmatrix} = \begin{pmatrix} \gamma_1 & -\beta_{21} \\ -\beta_{12} & \gamma_2 \end{pmatrix} \begin{pmatrix} n_1 - b_1 T \\ n_2 - b_2 T \end{pmatrix} \text{ and } n'_A = n_A - \alpha(n'_D + n_A) \quad (1)$$

Here, T is a time specifying the length of a burst. The correction parameters were determined with two separate samples of the dyes in which their concentrations were adjusted such that both samples have an absorbance of 0.1 at the excitation wavelength (485 nm) (8). Setting arbitrarily $\gamma_1 = 1$, we obtain $\gamma_2 = 1.12 \pm 0.09$, $\beta_{21} = 0.050 \pm 0.003$, and $\beta_{12} = 0.0021 \pm 0.0004$ over 5 years with 21 measurements of these correction factors. The probability of directly exciting the acceptor dye at 485 nm is given by $\alpha = \epsilon_A/(\epsilon_A + \epsilon_D)$ where ϵ_A and ϵ_D are the extinction coefficients of the dyes at the donor excitation wavelength of 485 nm. For our dye pair, $\alpha = 0.05$. Transfer efficiency histograms were computed with the fully corrected photon counts according to

$$E = \frac{n'_A}{n'_A + n'_D} \quad (2)$$

Importantly, the corrections (eq. 1) are already taken into account during burst identification. Bursts were identified from the measured photon traces following Eggeling *et al.*(6) and Hoffmann *et al.* (7). If $\Delta t_i = t_i - t_{i-1}$ is the inter-photon time between the ith photon and its predecessor, the photon i is retained if $\Delta t_i \leq \gamma_j(i)\ \Delta t_{max}$ (Δt_{max} = 100 μs) with $\gamma_j(i)$ being the correction factor of the ith photon detected in channel j = [1,2] (see eq. 1). The algorithm then proceeds to the next photon $i + 1$, stops after n photons once $\Delta t_{i+n} > \gamma_j(i+n)\ \Delta t_{max}$, and provides the total length of the burst by $T = t_{n-1} - t_{i-1}$. The resulting string of photons is now corrected via eq. 1 using estimated background rates b_1 and b_2. The initial guess of b_1 and b_2 is given by all detected photons in channel 1 and 2, respectively, divided by the total measurement time. A burst is then identified if $(n'_A + n'_D) > 100$. The photons belonging to this burst are removed from the photon trace and a new guess for b_1 and b_2 is computed based on the remaining photons. Subsequently, the burst search is performed again with updated background rates. This procedure converges after three iterations to constant background rates and a constant number of identified bursts.

Since the identified bursts also contain molecules for which the acceptor bleached during the transit trough the confocal spot, thus masking the true transfer efficiency, we further cleaned the FRET histograms from these events (9). For a burst with n'_D donor photons with the arrival times $t_{D,1} \dots t_{D,n'_D}$ and n'_A acceptor photons with the arrival times $t_{A,1} \dots t_{A,n'_A}$, the average arrival times are given by $\langle t_D \rangle = n'^{-1}_D \sum_i t_{D,i}$ and $\langle t_A \rangle = n'^{-1}_A \sum_i t_{A,i}$. The burst asymmetry is defined by $\alpha_{DA} = \langle t_D \rangle - \langle t_A \rangle$. If the acceptor dye bleaches, we clearly find $\alpha_{DA} > 0$. Taking shot noise into account, the distribution of α_{DA} has a standard deviation given by

$$\sigma_{DA} = \frac{T}{2\sqrt{3}}\left(\frac{1}{n'_D} + \frac{1}{n'_A}\right)^{1/2}. \quad (3)$$

To eliminate molecules with a bleached acceptor, we excluded all molecules for which $|\alpha_{DA}| > \sigma_{DA}$. In addition, only molecules containing active acceptor and donor dyes were included in the analysis. To this end, we computed the donor-acceptor stoichiometry (S) for each burst according to

$$S = \frac{n'_{DD} + n'_{DA}}{n'_{DD} + n'_{DA} + \gamma_{PIE}\ n_{AA}} \quad . \quad (4)$$

Here, γ_{PIE} is a correction factor to account for the different excitation intensities for donor and acceptor. Furthermore, the first subscript indicates the emission and the second subscript indicates the excitation. Only molecules with $S < 0.8$ were used for constructing smFRET histograms.

FRET histograms were fitted with a combination of empirical log-normal and Gaussian distributions (10). For binding experiments with unlabeled β-cat, the width and position of the FRET-peaks were fixed to minimize the number of free fitting

parameters and the area under the fitted histogram curve for each subpopulation was determined using numerical integration.

The affinities obtained with smFRET can be compared to those obtained with ITC for unlabeled E-cad (11) that report $\Delta H = -39.2 \pm 0.2\, kcal/mol$ and a binding entropy of $T_0 \Delta S = -29\, kcal/mol$ at 30°C. The dissociation constant at our experimental temperature of T = 23°C is therefore given by $K_D = e^{-(\Delta H - T\Delta S)/RT}$, where R is the ideal gas constant. Using error propagation to account for the reported error in the binding enthalpy, we obtain an affinity of $K_D = 9.5 \pm 3.2\, nM$.

For distance calculations based on the mean transfer efficiencies, the Förster radius measured in water $R_0^6(x)$ was corrected for the different refractive indices of the solutions according to:

$$R_0^6(x) = R_{0,0}^6 \left(\frac{n_0}{n(x)}\right)^4 \quad (5)$$

where $n(x)$ is the refractive index of the sample at condition x. Refractive indices were measured with an Abbe refractometer (Krüss) and were used to calculate the exact KCl concentrations (12, 13). The Foerster distance in water is 5.4 nm (14).

Two-focus FCS (2fFCS). 2fFCS measurements were conducted on a MicroTime200 (PicoQuant, Berlin) confocal microscope (Olympus). The light of two orthogonally polarized, pulsed diode lasers is combined with a polarization sensitive beam splitter and afterwards coupled into a polarization maintaining single-mode fiber. Both lasers (483 nm, LDH-D-C-485, PicoQuant) are pulsed alternately with a total repetition rate of 40 MHz and a laser power of 30 μW each. Before entering the objective, the laser beam passes through a Nomarski prism (U-DICTHC, Olympus). Afterwards, the two laser beams are focused by the objective thus causing two overlapping excitation volumes with a small lateral shift. Emission light passes through the objective, prism, and the dichroic beamsplitter (see section Single-molecule fluorescence spectroscopy) and is focused onto a pinhole (150 μm). Behind the pinhole the light is collimated and divided by a polarizing beam-splitter and focused onto two single-photon avalanche diodes (SPADs). For each laser focus, the corresponding autocorrelation function and the cross-correlation functions between foci were computed and fitted as described previously (15). To determine precise diffusion coefficients, we determined the distance between the two foci using the known Stokes radius of the dye Oregon Green and the water viscosity at the known lab-temperature of 23 ± 0.6°C. Fitting of the auto- and cross-correlation functions is performed as described by Dertinger *et al.*(15). We found a focal distance of 353 nm.

Determination of the affinity of the core-binding region of E-cad for β-cat. The core-binding region of E-cad with the highest contact probabilities (Fig. 5b)

consisted of a 20 amino acid stretch. Since smFRET is not suitable for monitoring conformational changes in such a short peptide, we used 2fFCS to determine the Stokes radius of the peptide at increasing concentrations of β-cat. To minimize that dye-labeling with AlexaFluor 488 massively impacted binding, we used two peptides in which the dye was either place at the N-terminus or at the C-terminus (SI Appendix Table S1). In addition, we introduced one serine-glycine repeat between the peptide and the cysteine used for labeling to increase the distance between the dye and the binding competent peptide. The free peptides had an average (average over both labeling constructs) Stokes radius of 1.5 ± 0.2 nm. At the highest β-cat concentration measured in our experiment, the Stokes radius was found to be 1.6 ± 0.2 nm, i.e., unchanged within the error. For comparison, the expected Stokes radius (16) of β-cat with a molecular weight of 85 kDa is 3.5 nm, which agrees with the Stokes radius of 3.9 nm found for donor-labeled E-cad in complex with β-cat (1 μM). The increased value in presence of E-cad is likely due to the flexible parts of E-cad that render the complex slightly larger than β-cat alone.

Temperature-controlled smFRET. Temperature-controlled smFRET experiments were conducted in a custom-built system. The design is very similar to a previous version(17, 18) and includes a temperature controlled sample holder and a second device to control the temperature of the objective. To precisely determine the temperature inside the confocal spot, we measured the diffusion coefficient of the dye Oregon Green (ThermoFisher Scientific) in water using 2f-FCS (15) (SI Appendix Fig. S7). The average of the fits from three independent measurements at each temperature in the range of 0-70 °C were used to determine the viscosity of water given the hydrodynamic radius of Oregon Green (0.6 nm) (19). The Stokes equation gives a relationship between the hydrodynamic radius R_S, the diffusion coefficient D, and the viscosity of the medium η:

$$\eta = \frac{k_B T}{6\pi D R_S} \tag{6}$$

where k_B is the Boltzmann constant and T the temperature. On the other hand, the water viscosity η has a known temperature dependence (20) given by

$$\eta = \eta_0 e^{ap + \frac{H-bp}{R(T-\theta-cp)}} \tag{7}$$

with p being the pressure in bar, T the temperature in K, and η_0, a, b, c, H and θ being constants ($E = 4.753\, kJ\, mol^{-1}$, $\eta_0 = 2.4055 \times 10\text{–}5\, Pa\, s$, $\theta = 139.7\, K$, $a = 4.42 \times 10^{-4}\, bar^{-1}$, $b = 9.565 \times 10^{-4}\, kJ\, mol^{-1} bar^{-1}$, and $c = 1.24 \times 10^{-2}\, K\, bar^{-1}$). When combining eq. 6 and 7, the temperature satisfies an implicit equation that relates the measured diffusion coefficient of Oregon Green to the

temperature of the sample, which was used to compute the actual temperature in the confocal spot (SI Appendix Fig. S7).

Determination of average donor-acceptor distances from FRET efficiencies. The mean FRET efficiency of all E-cad variants in the absence of β-cat were converted to distances according to (14)

$$\langle E \rangle = \frac{\int_0^L E(r)P(r)dr}{\int_0^L P(r)dr}\ . \quad (8)$$

Here, $E(r)$ is the well-known Foerster equation

$$E(r) = \frac{R_0^6}{R_0^6 + r^6} \quad (9)$$

with $R_0 = 5.4\,nm$. The conversion of mean transfer efficiencies to donor-acceptor distances (R_{DA}) is clearly model-dependent and we determined R_{DA} using eq. 8 with four polymer models: the Gaussian chain model (14), the Worm-like chain model (21), the Sanchez model(22-25), and the self-avoiding random walk model (SAW) (21, 26). The resulting R_{DA}-distances are all very similar. The model-dependent variation is shown as an error band in Fig. 2D.

Two-dimensional fluorescence lifetime vs. transfer efficiency plots. To identify whether the observed broadening of the FRET histograms arises from conformational heterogeneity, we computed two-dimensional plots that correlate the donor excited-state lifetime with the measured FRET efficiency (7). For a fixed distance r, the mean donor lifetime in the presence of acceptor is given by

$$\frac{\tau_{DA}(r)}{\tau_D} = 1 - E(r) \quad (10)$$

with τ_D being the lifetime of the donor in absence of the acceptor. Yet, in presence of a distribution of distances $P(r)$, the relationship between mean fluorescence lifetime and mean FRET efficiency changes. The mean fluorescence lifetime of the donor in the presence of an acceptor is then given by

$$\langle \tau_{DA} \rangle = \frac{\int_0^\infty tI(t)dt}{\int_0^\infty I(t)dt} \text{ with } I(t) = I_0 \int_0^\infty P(r) e^{-t/\tau_{DA}(r)} dr. \quad (11)$$

Here, $I(t)$ is the time-dependent fluorescence emission intensity. Simplifying equation (11), the average fluorescence lifetime is given by

$$\langle\tau_{DA}\rangle = \frac{\int_0^\infty \tau_{DA}(r)^2 P(r)dr}{\int_0^\infty \tau_{DA}(r)P(r)dr} \ . \qquad (12)$$

In Fig. 4B, we used the distance distribution for a Gaussian chain given by

$$P(r) = 4\pi r^2 \left(\frac{3}{2\pi\langle r^2\rangle}\right)^{\frac{3}{2}} e^{-\frac{3r^2}{2\langle r^2\rangle}} \qquad (13)$$

with $R_{DA} = \langle r^2\rangle^{\frac{1}{2}}$ being the average donor-acceptor distance to demonstrate the expected lifetime-FRET correlation for a fully disordered chain.

Polyampholyte theory. To describe the chain collapse with increasing concentrations of KCl, we fitted the change in donor-acceptor distance of all E-cad variants with a polyampholyte polymer theory developed by Higgs and Joanny (27)

$$\alpha^5 - \alpha^3 = \frac{4}{3}\left(\frac{3}{2\pi}\right)^{3/2} \sqrt{N}\nu \quad \text{with} \quad \alpha^2 = \frac{\langle r^2\rangle}{R_{ideal}^2} \qquad (14)$$

Here, $R_{ideal}^2 = Nb^2$ with N being the number of peptide bonds and $b = 0.38\,nm$, being the distance between two C_α-atoms. Expression (14) measures the deviation of the end-to-end distance with respect to the end-to-end distance of an ideal Gaussian chain. The two-body interaction term (excluded volume) ν has two contributions $\nu = \nu_0 + \nu_{el}$, non-electrostatic interactions (ν_0) and electrostatic interactions (ν_{el}). The latter is given by Higgs and Joanny as

$$\nu_{el} = \frac{4\pi l_B (f-g)^2}{b^3\kappa^2} - \frac{\pi l_B^2 (f+g)^2}{b^3\kappa}. \qquad (15)$$

Here, f and g are the fractions of positive and negative charges in the sequence of the individual E-cad variants, respectively. The Debye length is defined as $\kappa^{-1} = (8\pi l_B I)^{-\frac{1}{2}}$, where I is the ionic strength of the solution and l_B is the Bjerrum length, i.e., the distance at which the electrostatic energy of the interaction between two elementary charges equals thermal energy. We use the known relation $l_B = \frac{e^2}{4\pi\varepsilon_0\varepsilon_r k_B T}$, where e is the elementary charge, ε_0 is the permittivity of vacuum, ε_r is the dielectric constant, k_B is Boltzmann's constant, and T is the temperature. Hence, eq. 15 is fully determined by the protein sequence and the ionic strength of the medium. To describe the collapse at high concentrations of KCl, we assume a linear dependence between the non-electrostatic two-body interaction term $\nu_0 = c_1 + c_2[I]$ with I being the ionic strength of the solution and c_1 and c_2 are fitting constants (28).

Sequence charge decoration (SCD). We have used a variant of the sequence charge decoration (29, 30), SCD_{lowsalt},

$$SCD_{lowsalt} = \frac{1}{N}\sum_{i=2}^{N}\sum_{j=1}^{i-1} q_i q_j (i-j) \quad (16)$$

in which q_i and q_j are the charges of the i-th and j-th amino acids in the sequence. This metric is designed to predict the salt-induced conformational change of the intrinsically disordered proteins due to charge patterning. The chain will compact with addition of salt if SCD_{lowsalt} is positive, whereas increasing salt concentration will induce chain expansion if SCD_{lowsalt} is negative.

Nanosecond Fluorescence Correlation Spectroscopy (nsFCS). We compute subpopulation specific correlation functions. To this end, we are using sample concentrations of around 100-500 pM that still allow us to differentiate populations of molecules that exhibit different FRET efficiencies. In a first step, we identify the photon bursts from individual molecules as described above. For these bursts, the FRET efficiencies are determined and finally the correlation functions for the selected subset of molecules were computed. By distributing the photons onto two donor and two acceptor detectors, dead time and after-pulsing of the SPADs are avoided. The cross-correlation functions were fitted using

$$g(t) = N^{-1}\left[1 - c_{ab} e^{\left(-\frac{t-t_0}{\tau_{ab}}\right)}\right]\left[1 - c_c e^{\left(-\frac{t-t_0}{\tau_c}\right)}\right]\left[1 + c_T e^{\left(-\frac{t-t_0}{\tau_T}\right)}\right]. \quad (17)$$

The three terms in brackets describe photon antibunching (ab), conformational dynamics (c), and triplet blinking of the dyes (T). In addition, N is the effective number of molecules in the confocal volume and c_{ab} , c_c , and c_T are amplitudes. The correlation times τ_{ab} , τ_c , τ_T and the time origin t_0 are fitting parameters. To graphically compare different experiments, the correlation functions in Fig. 4A were normalized by N and the triplet term. The correlation times τ_c for the different E-cad constructs in the absence of β-cat are shown in Fig. 4F.

The nsFCS experiments with donor excitation result in positive donor-acceptor cross-correlation amplitudes for segments B, C, and BC in complex with β-catenin. This implies a correlated on-off behavior of both dyes, e.g., by static quenching of the donor via stacking complexes with aromatic amino acids such as tryptophan (31). Since the quenched donor in complex with an aromatic amino acid cannot transfer energy to the acceptor, the photon rate of both dyes will fluctuate in a concerted fashion thus causing the positive cross-correlation amplitude (31). It has also been shown that energy can be transferred from the unquenched donor to the quenched acceptor (31). Importantly, since the acceptor dye is chemically similar to the donor (both dyes are rhodamine derivatives) and since our samples contain both labeling permutants, i.e., each of the introduced cysteine residues is labeled with donor or acceptor, a quenching of the donor implies that also the acceptor can be quenched. To circumvent the

preparation of donor-only labeled samples for monitoring quenching in the absence of FRET, we used a direct excitation of the acceptor to monitor quenching dynamics in the same samples used for the smFRET experiments. The resulting auto-correlation functions were fitted using eq. 17 and the amplitude c_c is shown in SI Appendix Fig. S2.

Determining the intra-chain diffusion coefficient of E-cad ABC from nsFCS. We determined the intra-chain diffusion coefficient of free E-cad ABC by modeling its dynamics as a diffusive process in the potential of mean force given by the donor-acceptor distance distribution that resulted as a best fit from the CG-simulations (Fig. 6B inset). In this picture, the measured correlation time τ_c obtained from nsFCS experiments obeys a direct relationship to the intra-chain diffusion coefficient D_0 given by (32)

$$\tau_c = {D_0}^{-1} \int_0^L P(r)^{-1} \left[\int_0^r \delta n(\rho) P(\rho) d\rho \right] / \int_0^L \delta n(r)^2 P(r) dr \tag{18}$$

with

$$\delta n(r) = n(r) - \langle n \rangle, \tag{19}$$

where $P(r)$ is the donor-acceptor distance distribution determined from the CG-simulation and $n(r)$ is the donor photon rate. The latter is determined from the photophysics of the FRET system with donor (D) and acceptor (A) that includes the four states DA, D*A, D*A*, DA* where the asterisk indicates an excited state. The rate matrix for this system is given by (in the basis given above)

$$\mathbf{K} = \begin{pmatrix} -(1+\alpha)k_{ex} & k_D & 0 & k_A \\ k_{ex} & -\left(k_D + \alpha k_{ex} + k_T(r)\right) & k_A & 0 \\ 0 & \alpha k_{ex} & -(k_D + k_A) & k_{ex} \\ \alpha k_{ex} & k_T(r) & k_D & -(k_{ex} + k_A) \end{pmatrix} \tag{20}$$

where $k_{ex} = 0.018\, ns^{-1}$ is the donor excitation rate, $k_D = 0.28\, ns^{-1}$ is the donor emission rate, $k_A = 0.25\, ns^{-1}$ is the acceptor emission rate, and $\alpha = 0.05$ is the direct excitation probability. The energy transfer rate is given by $k_T(r) = k_D (R_0/r)^6$. The steady-state vector of the four states $\mathbf{p_{ss}}$ is the solution of the algebraic system $\mathbf{0} = \mathbf{K}\mathbf{p_{ss}}$ where $\mathbf{0}$ is the null vector. The photon rate $n(r)$ of the system is then given by $n(r) = \mathbf{1}^{\mathrm{T}}\mathbf{V}\mathbf{p_{ss}}$ where

$$\mathbf{V} = k_D \begin{pmatrix} 0 & 1 & 0 & 0 \\ 0 & 0 & 0 & 0 \\ 0 & 0 & 0 & 0 \\ 0 & 0 & 1 & 0 \end{pmatrix} \tag{21}$$

is the donor photon detection matrix. We determined the intra-chain diffusion coefficient $D_0 = 42 \pm 1\ nm^2/\mu s$ of free E-cad ABC (Fig. 6C) by numerically solving eq. 18.

Recurrence analysis of single particles (RASP). The recurrence analysis of single particles (RASP) uses the fact that a freely diffusing molecule can be observed multiple times in a single-molecule experiment. Once a molecule leaves the observation volume, the chance of it returning to the confocal spot is greater than the chance of detecting a new molecule for short time intervals (SI Appendix Fig. S4A-B). To extract kinetics based on this effect, we binned our data in 100 µs bins and computed the bin-time autocorrelation function $G(\tau)$ that contains the information of the likelihood to observe two signal-containing bins separated by the delay time τ

$$G(\tau) = \frac{p(\{b_1, t_1\}, \{b_2, t_1 + \tau\})}{p(\{b_1, t_1\}, \{b_2, t_1\})} \tag{22}$$

Here, $p(\{b_1, t_1\}, \{b_2, t_1 + \tau\})$ denotes the joint probability of observing two bins $b_1$and b_2 at times t_1 and $t_1 + \tau$, respectively, and $p(\{b_1, t_1\})$ and $p(\{b_2, t_1\})$ are the probabilities of detecting $b_1$and b_2, respectively, at time t_1. This correlation function can be converted to $P_{same}(\tau)$, i.e., the probability that bins separated by the time τ originate from the same molecule (33) (SI Appendix Fig. S4B):

$$P_{same}(\tau) = 1 - \frac{1}{G(\tau)}\ . \tag{23}$$

During the time τ between detecting bin 1 (b_1) and bin 2 (b_2), the molecule can change its conformation, thus causing a different FRET efficiency in b_2. To quantify this effect, we define two regions of the broad FRET histograms of the E-cad/β-cat complex, region 1 and region 2 (SI Appendix Fig. S4C). In a first step, we select all bins with FRET efficiencies in region 1. For each of these selected bins, we then identify the bins that follow in time. To this end, we create a 100 µs time window and move it in steps of 100 µs (time delay τ) along the experimental time trace. At each of these time steps, we determine the FRET efficiency of the bins in the window and construct a FRET histogram, which is called the recurrence histogram. The procedure results in a time series of the recurrence histograms, one for each delay time (SI Appendix Fig. S4D). We repeat this procedure with molecules in region 2 instead of region 1, which results in a second time series of recurrence histograms (SI Appendix Fig. S4D). At very short time delays, the two series of recurrence histograms will

reflect the FRET-efficiencies of the initially selected bins, i.e., that of region 1 for series 1 and region 2 for series 2. Yet, at longer time delays, the recurrence histograms will change due to (i) a change in conformation and (ii) because new molecules entered the confocal volume. To identify this change in the recurrence FRET-histograms for different time delays, the histograms were globally fitted with a sum of a log-normal function for molecules that lack an active acceptor dye (E = -0.05) and two Gaussian functions for the populations from regions 1 and 2. The positions, widths, and asymmetries were determined from the histograms of both regions at the initial time window, i.e., at $\boldsymbol{\tau} = 0$. The relative populations of the molecules in region 1 and region 2 were obtained by integrating the respective sub-populations. SI Appendix Fig. S4E depicts the time course of the fraction of molecules in region 1 after originally selecting molecules in region 1 (blue) or molecules in region 2 (red), respectively. By increasing the time delay τ, the fraction of molecules in region 1 when initially selecting region 1 decrease and those in region 1 when selecting region 2 increase. The observed decays $P_m(\tau)$ are a combination of two contributions: conformational switching between the two selected regions, characterized by $P_{conf}(\tau)$, and the time-dependent likelihood that two observed bins or "snapshot" arise from the same molecule, $P_{same}(\tau)$. Since we can compute $P_{same}(\tau)$ directly from the burst time autocorrelation function of our data, we can determine $P_{conf}(\tau)$, the actual kinetics of conformational switching. Thus, the observed increase in the population of molecules in region 1 in the recurrence histograms after initially selecting molecules in region 2 is given by

$$P_m(\tau) = P_{same}(\tau)P_{conf}(\tau) + [1 - P_{same}(\tau)]\rho_{eq}. \qquad (24)$$

Here, $P_m(\tau)$ is the measured time decay, $P_{same}(\tau)$ is the probability that two bins separated by the time delay τ are from the same molecule, $P_{conf}(\tau)$ is the probability that the FRET efficiency of a molecule changes during τ due to conformational dynamics, $1 - P_{same}(\tau)$ is the probability that two bins separated by the time delay τ are from different molecules, and ρ_{eq} is the *a priori* probability that a molecule exhibits a FRET efficiency from region 1 (or 2), which is given by the equilibrium FRET histogram. For a better visualization and easier analysis, we directly determine the 'true' kinetics of switching $P_{conf}(\tau)$ that we obtain by re-arranging equation (24) for $P_{conf}(\tau)$. In addition, we average the two kinetic traces, i.e., the increase of molecules in region 1 after initial selection of molecules from region 2 and the depletion of molecules in region 1 after initial selection of molecules from region 1. The resulting averaged traces are shown in Fig. 4c. The RASP decays were fit with an exponential function $P_{conf}(\tau) = a\, e^{-t/\tau_r} + b$ where a is the amplitude, b is the offset, and τ_r is the relaxation time (Fig. 4E). The determined relaxation times are shown in Fig. 4F.

Arrhenius fit of the RASP relaxation times. We fit the RASP relaxation times for the complex between the E-cadherin ABC construct and b-catenin using the standard Arrhenius equation

$$\tau_r = Ae^{\beta E_a} \tag{25}$$

where $A = 3.5\ 10^{-5}\ \pm 2\ .3\ 10^{-4}\ ms$ and $E_a = 28\ \pm 16\ kJ/mol$. Notably, the fitting error of the pre-factor A is enormous. To check whether the change in relaxation times with increasing temperature can be explained solely by a change in the water viscosity, we also fit the data using

$$\tau_r = A\eta(T) \tag{26}$$

where the temperature-dependent viscosity $\eta(T)$ is given by eq. 7 and A is a constant. We obtained a good fit of the data with $A = 0.586\ \pm 0.061\ mPa^{-1}$.

Fitting RASP kinetics by numerically solving the Smoluchowski equation. As an alternative to exponential fitting, we also fitted the RASP kinetics of the variant E-cad ABC bound to β-cat by solving the Smoluchowski equation

$$\partial u(t,r)/\partial t = D\,\partial/\partial r\,[V'(r) + \partial/\partial r]u(t,r) \tag{27}$$

with $u(t,r)$ being the time-dependent donor-acceptor distance distribution and $V(r) = -\ln P(r)$ being the potential of mean force with $P(r)$ being the normalized equilibrium donor-acceptor distance distribution obtained as a best fit from the CG-model (Fig. 6B inset). Equation 27 was solved with the boundary conditions

$$u(0,r) = \begin{cases} 0 & 0 \le r < r_b \\ Z(r_b)^{-1}P(r) & r_b \le r \le r_{max} \end{cases} \text{ with } u(t,0) = 0 \text{ and } u(t,r_{max}) = 0. \tag{28}$$

Here, $Z(r_b)$ is a normalization factor, r_b defines the distance that separates high-FRET (region 2) $(r < r_b)$ from low-FRET (region 1) $(r \ge r_b)$ molecules, and r_{max} is the upper integration limit (20 nm). An analytical expression for $P(r)$ suitable to efficiently solve eq. 27 was obtained by fitting the distance distribution for E-cad ABC from the CG-model with the superposition of three Gaussian functions. The fraction of low-FRET molecules $f(t)$, i.e., molecules from region 1, was obtained from

$$f(t) = \int_{r_b}^{r_{max}} u(t,r)\,dr \Big/ \int_0^{r_{max}} u(t,r)\,dr. \tag{29}$$

The model contains two free parameters, the diffusion coefficient D and the separation distance r_b. The latter quantity was determined prior to fitting by numerically solving

$$\langle f\rangle = \int_{r_b}^{r_{max}} P(r)\,dr \Big/ \int_{0}^{r_{max}} P(r)\,dr \tag{30}$$

for r_b where $\langle f\rangle$ is the average fraction of the last 2 ms of the kinetic time traces, i.e., from 8 – 10 ms. The resulting value for r_b was then used for fitting. We performed an error estimation of the diffusion coefficient (D) using bootstrapping the kinetic time traces with $n = 3$. We then described the temperature dependence of the diffusion coefficient (Fig. 6C) as

$$D = D_0 e^{-\beta^2\sigma^2} \text{ or } D = D_0 e^{-2\beta\sigma} \tag{31}$$

where the first expression corresponds to Gaussian distributed roughness and the second term for periodic roughness along a one-dimensional free energy landscape (34). The value $D_0 = 42\ \pm 1\ nm^2/\mu s$ was obtained from the nsFCS experiments (eq. 18-21). To account for the change in water viscosity at increasing temperatures in eqs 31, we assumed

$$D_0 = \frac{aT}{\eta(T)} \tag{32}$$

and determined the factor a using the known value $D_0 = 42\ \pm 1\ nm^2/\mu s$ at the temperature $T = 296\,K$ and viscosity given by eq. 7. We obtained $a = 147\ Pa\ nm^2/K$. Replacing D_0 in eqs. 31 by the expression in eq. 32 then leaves the roughness value σ as a single fitting parameter.

Molecular simulations with the CG-model. We parameterized a coarse-grained (CG) model to characterize the interactions between E-cad and β-cat. The model was based on our recently developed HPS model(35) in which each amino acid was represented as a bead. The potential energy can be written as,

$$E = E_{\text{bonded}} + E_{\text{electrostatic}} + E_{\text{IDP}}(\epsilon) + E_{\text{complex}}(\zeta), \tag{29}$$

in which there are three types of interactions for disordered E-cad: bonded, electrostatic and short-range pairwise interactions, and one additional term for the interactions between E-cad and β-cat. The bonded interactions were modeled by a harmonic potential with a bond length of 3.8 Å and a spring constant of 10 kJ/Å^2. The electrostatic interactions were modeled with a Coulombic term using Debye-Hückel electrostatic screening (36). The short-range pairwise interactions were modeled using Ashbaugh-Hatch functional form (37) and the depths of the potential minimums are proportional to the amino acid hydrophobicity values (λ) (38). The free parameter ε, which was originally tuned according to the size of the IDPs, has been adjusted to

0.16 kcal/mol using the experimental FRET efficiencies of the six E-cad segments (SI Appendix Fig. S8). To simulate β-cat in the CG model, we represented the structured part of β-cat in the experimentally solved structure (PDB: 1i7x)(39) as a rigid body in the simulation. The interactions between E-cad and β-cat were also modeled based on the native interactions in the same structure. A native interaction was assumed if the Cα distance between two amino acids is smaller than 1.2 times the sum of the amino acid radii used in the CG model. For every pair of amino acids that form native interactions in the complex, we have introduced one rigid and one flexible CG-models, with different types of potential energy functions. First for the rigid model, a harmonic potential energy function with the native interaction distance as the minimum position and a large spring constant of 20 kcal/mol were introduced. This effectively rigidified all the native contacts as seen in both X-ray structures. Second for the flexible model, a 12-6 Leonard-Jones (LJ) potential energy function with the native interaction distance as the minimum position was introduced. The depth of the LJ-well, i.e., the interaction strength ζ was adjusted to 0.6 kcal/mol according to the FRET efficiencies of the six segments of E-cad in the complex at an ionic strength of 82 mM. The deviations to the experimental FRET efficiencies for both models were shown in SI Appendix Fig. S8. The CG simulation of E-cad alone was run for 10 μs and the complex simulation was run for 20 μs, using LAMMPs(40) and HOOMD-Blue(41) to benefit from both CPU and GPU resources. The first 1 μs of the simulation was always excluded from the analysis. To obtain the FRET efficiencies from the simulations, the linker of both dyes were taken to be equivalent 9 amino acids(42), i.e., 4.5 amino acids per linker.

Rouse model with surface interactions. Here we show that for a simple bead-and-spring model, contacts with a surface increase the effective spring constant. In one dimension, the Langevin equation for the coordinate of the kth bead ($x_k = x_k(t)$) that contacts its neighbour beads is well known (43, 44). All beads also contact a specific location on a surface ($y = 0$), which, for simplicity, is identical for all beads and does not fluctuate in time

$$\xi \dot{x}_k(t) + k_1\big(2x_k(t) - x_{k-1}(t) - x_{k+1}(t)\big) + k_2 x_k(t) = X_k(t) \tag{30}$$

Here, ξ is a friction coefficient and k_1 and k_2 are spring constants for bonds in the chain and with the surface, respectively. X_k is Gaussian distributed white noise. Assuming a circular chain(44), taking the Fourier transform in bead number ($k \to q$) and time ($t \to \omega$), and rearranging, we obtain

$$x''_q(\omega) = X''_q(\omega)/[-i\omega\xi + 2k_1(1 - \cos q) + k_2]. \tag{31}$$

The relaxation time for the largest modes of such a chain is then given by $\tau_q = \xi / k_{eff}$ with the effective spring constant $k_{eff} = k_1 q^2 + k_2$, showing that the spring constant of a chain is affected by surface interactions.

SI Figures

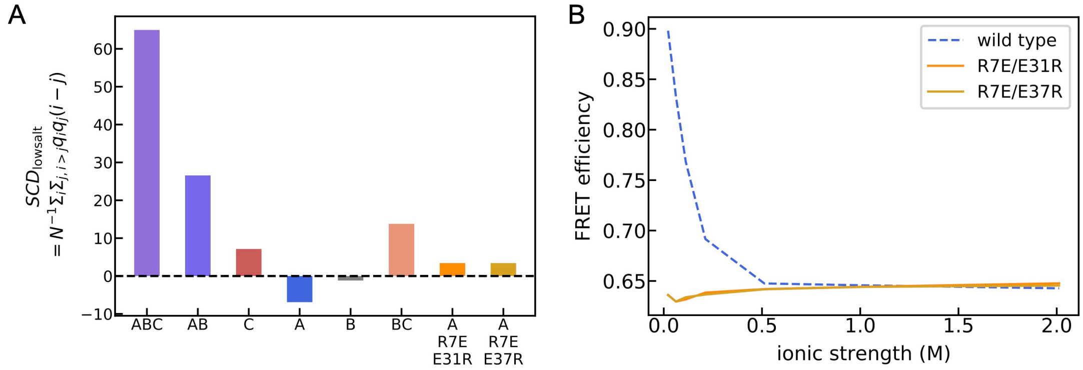

Fig. S1: Sequence charge decoration metric ($SCD_{lowsalt}$, see Methods) for all E-cad segments computed based on the amino acid sequence (**A**) and salt-dependent FRET efficiencies of wild type E-cad and two charge-swapped variants of the A-segment (**B**).

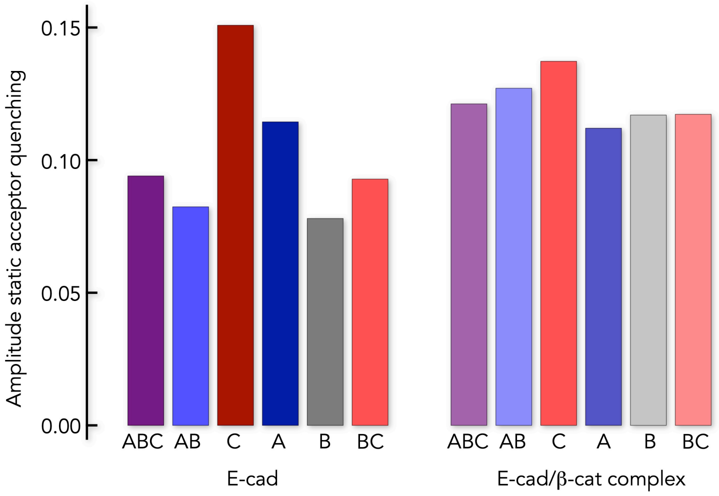

Fig. S2. nsFCS of all E-cad variants in the absence (left) and presence (right) of β-cat after exciting the acceptor directly. Quantitative comparison of the quenching amplitudes show that the C-segment has the highest amplitude in free E-cad. All quenching amplitudes are elevated in complex with β-cat.

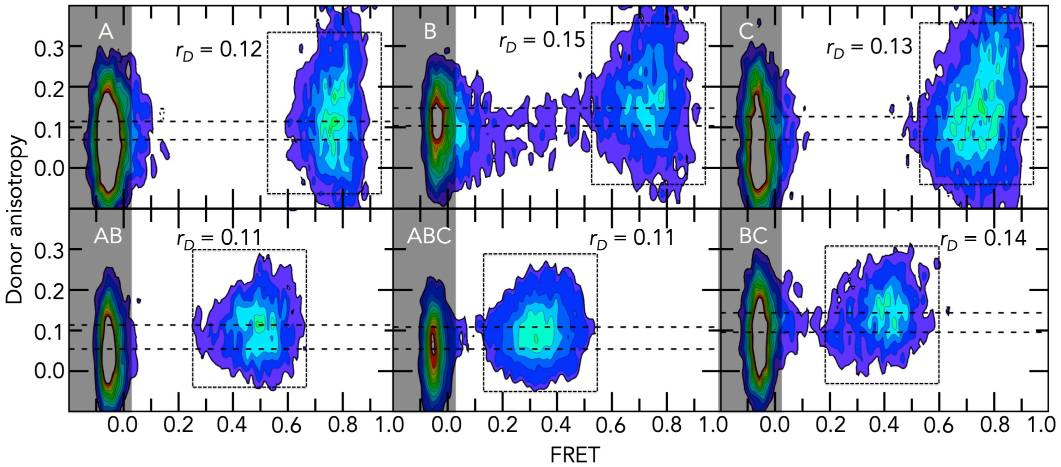

Fig. S3. Steady-state donor anisotropy of the six E-cad variants (indicated) in complex with β-cat. The population at a FRET efficiency close to zero arises from molecules that lack an active acceptor (gray shaded area). The second population at higher FRET efficiency (box) results from E-cad molecules with donor and acceptor in complex with β-cat. For all variants, the steady-state anisotropies are between 0.1 and 0.2, indicating moderate restriction in the rotational freedom of the donor dye. The average donor anisotropies of the FRET-population is indicated.

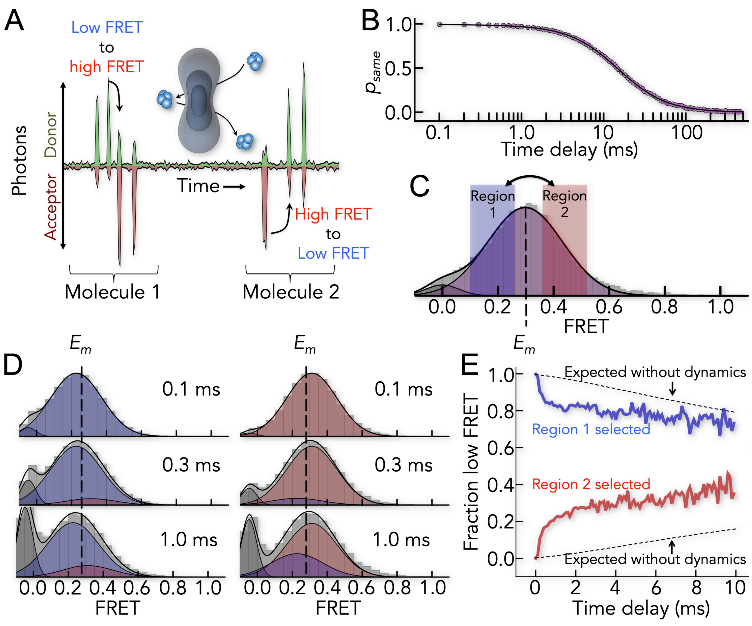

Fig. S4. Recurrence analysis of single particles (RASP) of the E-cad/β-cat complex. (**A**) Scheme of single molecules that are detected while they diffuse through the confocal volume. After the initial molecule exits the confocal volume, it can either return or a new molecule can enter. (**B**) Time-dependent probability that two molecules detected with a time delay are the same is decaying with increasing time between their detection. (**C**) Equilibrium FRET histogram of the E-cad/β-cat complex. For RASP, molecules were selected either from region 1 (low FRET) or from region 2 (high FRET). The dashed line indicates the mean FRET position (E_m). (**D**) Time-dependent recurrence histograms of molecules selected from region 1 (left) and region 2 (right). Solid lines are fits with a superposition of Gaussian peaks. The dashed line indicates the mean FRET position (E_m) of the equilibrium FRET histogram shown in C. (**E**) Time decays of the fraction of molecules in region 1 (low-FRET) after initially selecting molecules from region 1 (blue) or region 2 (red). The dashed line indicates the expected kinetics in the absence of conformation dynamics as computed from the p_{same} (see panel B).

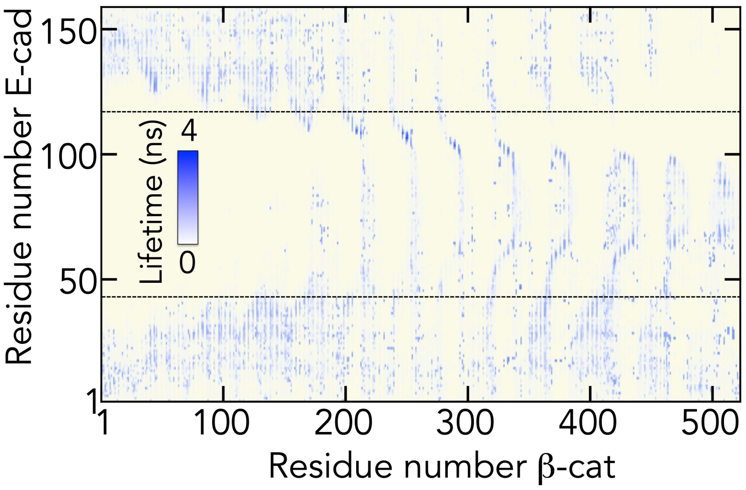

Fig. S5. Contact lifetimes in the E-cad/β-cat complex from the flexible CG-model. The contact lifetime map shows a significant distribution of contacts across the whole β-cat surface.

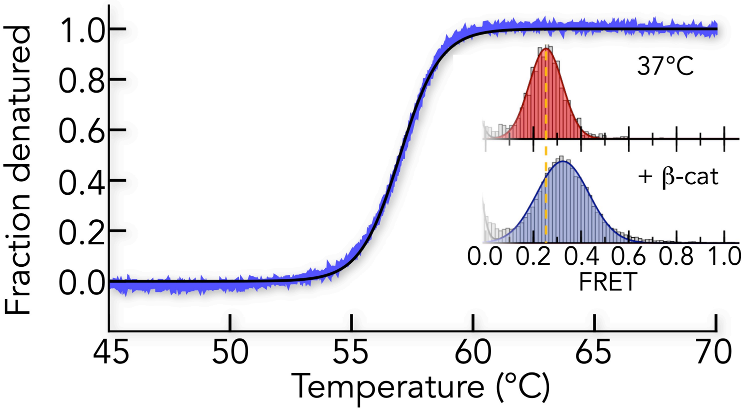

Fig. S6. Thermal stability of β-cat monitored with nano-differential scanning fluorimetry (nanoDSF). The melting curve shows a denaturation at 57°C, indicating that β-cat is stable at the highest temperature used in the temperature-dependent RASP experiments (main text Fig. 6). Inset: SmFRET histograms of E-cad ABC at 37°C in the absence (red) and presence (blue) of β-cat (200 nM). The significant broadening of the FRET-distribution at 37°C and the shift to higher FRET-values indicate binding of E-cad at 37°C. Solid lines are fits with a superposition of two Gaussian peaks.

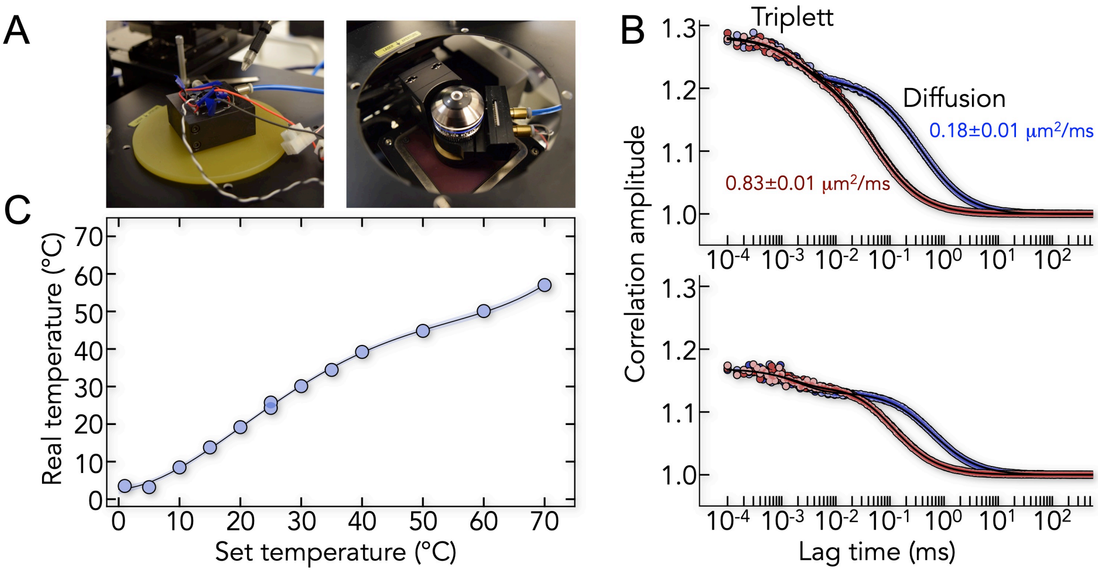

Fig. S7. Calibration of smFRET experiments at different temperatures. (**A**) Peltier-controlled sample holder (left) and device to control the temperature of the objective (right). (**B**) Dual-focus FCS autocorrelation functions (top) and cross-correlation functions (bottom) of the dye Oregon Green at set temperatures 0°C (blue circles) and 70°C (red circles). Solid lines are fits as described in the Methods section. The diffusion coefficients at the two temperatures are indicated. (**C**) Temperature calibration curve obtained by determining the diffusion coefficient of Oregon Green using 2fFCS. The x-axis shows the temperature set on the sample controller. The objective controller is set to the same temperature up to a set-temperature of 40°C. For higher temperatures, the objective is kept at a set-temperature of 40°C. The y-axis shows the temperature determined based on the diffusion coefficient of Oregon Green and the known temperature dependence of the water viscosity (see Methods). The solid line is a polynomial fit of fourth order to the data and the blue shaded are indicates the 90% confidence band.

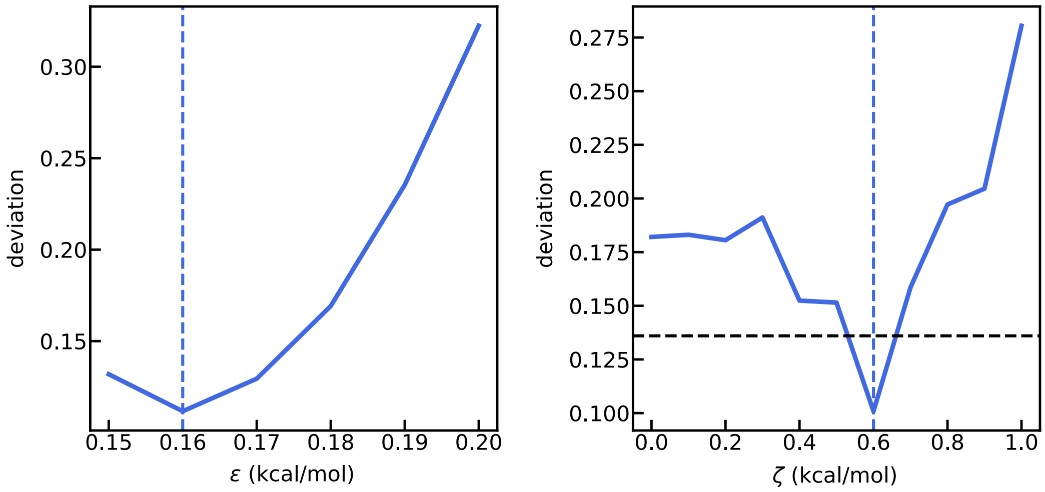

Fig. S8. Parameterization of the coarse-grained (CG) model. Left: Deviation of the calculated FRET-values from the experimental data of the six constructs when varying the interaction strength (ε) of E-cad only. Right: The deviation of the model from the FRET experiment of the six constructs at the experimental ionic strength of 82 mM when varying the interaction strength (ζ) between E-cad and β-cat for the flexible CG-model (see Methods). The blue dashed lines indicate the minimum and the black dashed lines indicate the deviation for the rigid CG-model.

Table S1. E-cad variants and sequence of β-cat used in the smFRET experiments. Phosphorylation sites as identified in ref **(39)** are indicated in yellow.

**ABC**	ACSGLRRRTVVKEPLLPPDDDTRDNVYYYDEEGGGEEDQDFDLSQLHRGLDARPEVTRNDVAPTLMSVPQYRPRPAN<br>PDEIGNFIDENLKAADSDPTAPPYDSLLVFDYEGSGSEAASLSSLNSSESDQDQDYDYLNEWGNRFKKLADMYGGGE<br>DDGSC
**AB**	ACSGLRRRTVVKEPLLPPDDDTRDNVYYYDEEGGGEEDQDFDLSQLHRGLDARPEVTRNDVAPTLMSVPQYRPRPAN<br>PDEIGNFIDENLKAADSDPTAPPYDSLLVFDYEGSGSEAACLSSLNSSESDQDQDYDYLNEWGNRFKKLADMYGGGE<br>DD
**BC**	ALRRRTVVKEPLLPPDDDTRDNVYYYDEEGGGEEDQDFDLCQLHRGLDARPEVTRNDVAPTLMSVPQYRPRPAN<br>PDEIGNFIDENLKAADSDPTAPPYDSLLVFDYEGSGSEAASLSSLNSSESDQDQDYDYLNEWGNRFKKLADMYGGGE<br>DDGSC
**A**	ACSGLRRRTVVKEPLLPPDDDTRDNVYYYDEEGGGEEDQDFDLCQLHRGLDARPEVTRNDVAPTLMSVPQYRPRPAN<br>PDEIGNFIDENLKAADSDPTAPPYDSLLVFDYEGSGSEAASLSSLNSSESDQDQDYDYLNEWGNRFKKLADMYGGGE<br>DD
**B**	ALRRRTVVKEPLLPPDDDTRDNVYYYDEEGGGEEDQDFDLCQLHRGLDARPEVTRNDVAPTLMSVPQYRPRPAN<br>PDEIGNFIDENLKAADSDPTAPPYDSLLVFDYEGSGSEAACLSSLNSSESDQDQDYDYLNEWGNRFKKLADMYGGGE<br>DD
**C**	ALRRRTVVKEPLLPPDDDTRDNVYYYDEEGGGEEDQDFDLSQLHRGLDARPEVTRNDVAPTLMSVPQYRPRPAN<br>PDEIGNFIDENLKAADSDPTAPPYDSLLVFDYEGSGSEAACLSSLNSSESDQDQDYDYLNEWGNRFKKLADMYGGGE<br>DDGSC
**Core 1**	CSGAADSDPTAPPYDSLLVFDYE
**Core 2**	AADSDPTAPPYDSLLVFDYEGSC
**β-cat**	ATQADLMELDMAMEPDRKAAVSHWQQQSYLDSGIHSGATTTAPSLSGKGNPEEEDVDTSQVLYEWEQGFSQSFTQEQ<br>VADIDGQYAMTRAQRVRAAMFPETLDEGMQIPSTQFDAAHPTNVQRLAEPSQMLKHAVVNLINYQDDAELATRAIPE<br>LTKLLNDEDQVVVNKAAVMVHQLSKKEASRHAIMRSPQMVSAIVRTMQNTNDVETARCTAGTLHNLSHHREGLLAIF<br>KSGGIPALVKMLGSPVDSVLFYAITTLHNLLLHQEGAKMAVRLAGGLQKMVALLNKTNVKFLAITTDCLQILAYGNQ<br>ESKLIILASGGPQALVNIMRTYTYEKLLWTTSRVLKVLSVCSSNKPAIVEAGGMQALGLHLTDPSQRLVQNCLWTLR<br>NLSDAATKQEGMEGLLGTLVQLLGSDDINVVTCAAGILSNLTCNNYKNKMMVCQVGGIEALVRTVLRAGDREDITEP<br>AICALRHLTSRHQEAEMAQNAVRLHYGLPVVVKLLHPPSHWPLIKATVGLIRNLALCPANHAPLREQGAIPRLVQLL<br>VRAHQDTQRRTSMGGTQQQFVEGVRMEEIVEGCTGALHILARDVHNRIVIRGLNTIPLFVQLLYSPIENIQRVAAGV<br>LCELAQDKEAAEAIEAEGATAPLTELLHSRNEGVATYAAAVLFRMSEDKPQDYKKRLSVELTSSLFRTEPMAWNETA<br>DLGLDIGAQGEALGYRQDDPSYRSFHSGGYGQDALGMDPMMEHEMGGHHPGADYPVDGLPDLGHAQDLMDGLPPGDS<br>NQLAWFDTDL

SI References

1. Erijman A, Dantes A, Bernheim R, Shifman JM, & Peleg Y (2011) Transfer-PCR (TPCR): a highway for DNA cloning and protein engineering. *J Struct Biol* 175(2):171-177.
2. Unger T, Jacobovitch Y, Dantes A, Bernheim R, & Peleg Y (2010) Applications of the Restriction Free (RF) cloning procedure for molecular manipulations and protein expression. *J Struct Biol* 172(1):34-44.
3. Frey S & Goerlich D (2014) Purification of protein complexes of defined subunit stoichiometry using a set of orthogonal, tag-cleaving proteases. *J Chromatogr A* 1337:106-115.
4. Müller BK, Zaychikov E, Bräuchle C, & Lamb DC (2005) Pulsed interleaved excitation. *Biophys J* 89(5):3508-3522.
5. Kapanidis AN*, et al.* (2005) Alternating-laser excitation of single molecules. *Acc Chem Res* 38(7):523-533.
6. Eggeling C*, et al.* (2001) Data registration and selective single-molecule analysis using multi-parameter fluorescence detection. *J Biotechnol* 86(3):163-180.
7. Hoffmann A*, et al.* (2007) Mapping protein collapse with single-molecule fluorescence and kinetic synchrotron radiation circular dichroism spectroscopy. *Proc Natl Acad Sci USA* 104(1):105-110.
8. Schuler B (2007) Application of single molecule Förster resonance energy transfer to protein folding. *Methods Mol Biol* 350:115-138.
9. Hillger F, Nettels D, Dorsch S, & Schuler B (2007) Detection and analysis of protein aggregation with confocal single molecule fluorescence spectroscopy. *J Fluoresc* 17(6):759-765.
10. Benke S, Nettels D, Hofmann H, & Schuler B (2017) Quantifying kinetics from time series of single-molecule Förster resonance energy transfer efficiency histograms. *Nanotechnology* 28(11):114002.
11. Choi H-J, Huber AH, & Weis WI (2006) Thermodynamics of β-Catenin-Ligand Interactions THE ROLES OF THE N- AND C-TERMINAL TAILS IN MODULATING BINDING AFFINITY. *J Biol Chem* 281(2):1027-1038.
12. Haynes WM (2014) *Handbook of Chemistry and Physics* (CRC Press) 95 Ed.
13. Pace CN (1986) Determination and analysis of urea and guanidine hydrochloride denaturation curves. *METHOD ENZYMOL* 131:266-280.
14. Schuler B, Lipman E, & Eaton W (2002) Probing the free-energy surface for protein folding with single-molecule fluorescence spectroscopy. *Nature* 419(6908):743-747.
15. Dertinger T*, et al.* (2007) Two-focus fluorescence correlation spectroscopy: a new tool for accurate and absolute diffusion measurements. *Chemphyschem* 8(3):433-443.
16. Wilkins D*, et al.* (1999) Hydrodynamic radii of native and denatured proteins measured by pulse field gradient NMR techniques. *Biochemistry* 38(50):16424-16431.
17. Aznauryan M, Nettels D, Holla A, Hofmann H, & Schuler B (2013) Single-molecule spectroscopy of cold denaturation and the temperature-induced collapse of unfolded proteins. *J Am Chem Soc* 135(38):14040-14043.
18. Nettels D*, et al.* (2009) Single-molecule spectroscopy of the temperature-induced collapse of unfolded proteins. *Proc Natl Acad Sci USA* 106:20740-20745.
19. Russinova E, Tretyachenko-Ladokhina V, Vele OE, Senear DF, & Alexander Ross JB (2002) Alexa and Oregon Green dyes as fluorescence anisotropy probes for measuring protein–protein and protein–nucleic acid interactions. *Anal Biochem* 308(1):18-25.

20. Likhachev ER (2003) Dependence of water viscosity on temperature and pressure. *Tech. Phys.* 48(4):514-515.
21. O'Brien EP, Morrison G, Brooks BR, & Thirumalai D (2009) How accurate are polymer models in the analysis of Förster resonance energy transfer experiments on proteins? *J Chem Phys* 130(12):124903.
22. Sanchez I (1979) Phase Transition Behavior of the Isolated Polymer Chain. *Macromolecules* 12:980-988.
23. Hofmann H*, et al.* (2012) Polymer scaling laws of unfolded and intrinsically disordered proteins quantified with single-molecule spectroscopy. *Proc Natl Acad Sci USA* 109(40):16155-16160.
24. Ziv G & Haran G (2009) Protein folding, protein collapse, and tanford's transfer model: lessons from single-molecule FRET. *J Am Chem Soc* 131(8):2942-2947.
25. Sherman E & Haran G (2006) Coil-globule transition in the denatured state of a small protein. *Proc Natl Acad Sci U S A* 103(31):11539-11543.
26. Zheng W*, et al.* (2018) Inferring properties of disordered chains from FRET transfer efficiencies. *J Chem Phys* 148(12):123329.
27. Higgs PG & Joanny J-F (1991) Theory of polyampholyte solutions. *J Chem Phys* 94(2):1543-1554.
28. Vancraenenbroeck R, Harel YS, Zheng W, & Hofmann H (2019) Polymer effects modulate binding affinities in disordered proteins. *Proc Natl Acad Sci USA* 116(39):19506-19512.
29. Sawle L & Ghosh K (2015) A theoretical method to compute sequence dependent configurational properties in charged polymers and proteins. *J Chem Phys* 143(8):085101.
30. Huihui J, Firman T, & Ghosh K (2018) Modulating charge patterning and ionic strength as a strategy to induce conformational changes in intrinsically disordered proteins. *J Chem Phys* 149(8):085101.
31. Haenni D, Zosel F, Reymond L, Nettels D, & Schuler B (2013) Intramolecular Distances and Dynamics from the Combined Photon Statistics of Single-Molecule FRET and Photoinduced Electron Transfer. *J Phys Chem B* 117(42):13015-13028.
32. Gopich IV, Nettels D, Schuler B, & Szabo A (2009) Protein dynamics from single-molecule fluorescence intensity correlation functions. *J Chem Phys* 131(9):095102.
33. Hoffmann A*, et al.* (2011) Quantifying heterogeneity and conformational dynamics from single molecule FRET of diffusing molecules: recurrence analysis of single particles (RASP). *Phys Chem Chem Phys* 13(5):1857-1871.
34. Zwanzig R (1988) Diffusion in a rough potential. *Proc Natl Acad Sci USA* 85(7):2029-2030.
35. Dignon GL, Zheng W, Kim YC, Best RB, & Mittal J (2018) Sequence determinants of protein phase behavior from a coarse-grained model. *PLoS Comput Biol* 14(1):e1005941.
36. Debye P & Hückel E (1923) Zur Theorie der Elektrolyte: I. Gefrierpunktserniedrigung und verwandte Erscheinungen. *Phys Z* 24(9):185-206.
37. Ashbaugh HS & Hatch HW (2008) Natively unfolded protein stability as a coil-to-globule transition in charge/hydropathy space. *J Am Chem Soc* 130(29):9536-9542.
38. Kapcha LH & Rossky PJ (2014) A simple atomic-level hydrophobicity scale reveals protein interfacial structure. *J Mol Biol* 426(2):484-498.
39. Huber AH & Weis WI (2001) The structure of the beta-catenin/E-cadherin complex and the molecular basis of diverse ligand recognition by beta-catenin. *Cell* 105(3):391-402.

40. Plimpton S (1995) Fast Parallel Algorithms for Short-Range Molecular Dynamics. *J Comput Phys* 117(1):1-19.
41. Anderson JA, Glaser J, & Glotzer SC (2020) HOOMD-blue: A Python package for high-performance molecular dynamics and hard particle Monte Carlo simulations. *Comput Mater Sci* 173:109363.
42. McCarney ER*, et al.* (2005) Site-specific dimensions across a highly denatured protein; a single molecule study. *J Mol Biol* 352(3):672-682.
43. Makarov DE (2010) Spatiotemporal correlations in denatured proteins: The dependence of fluorescence resonance energy transfer (FRET)-derived protein reconfiguration times on the location of the FRET probes. *J Chem Phys* 132(3):035104.
44. Soranno A, Zosel F, & Hofmann H (2018) Internal friction in an intrinsically disordered protein-Comparing Rouse-like models with experiments. *J Chem Phys* 148(12):123326.
